# Italian still life paintings as a resource for reconstructing past Mediterranean aquatic biodiversity

**DOI:** 10.1038/s44185-025-00103-8

**Published:** 2025-09-02

**Authors:** L. Merquiol, A.-S. Tribot, D. Faget, G. P. J. Denys, T. Richard, T. Changeux

**Affiliations:** 1 Aix Marseille Université, Université de Toulon, CNRS, IRD, MIO, ITEM, Marseille, France; 2 UMR TELEMMe, MMSH, Aix-Marseille University, CNRS, Aix-en-Provence, France; 3 Unité d’Appui à la Recherche Patrimoine Naturel – Centre d’expertise et de données (UMS 2006 OFB, MNHN, CNRS, IRD), Muséum national d’Histoire naturelle, Paris, France

**Keywords:** Biodiversity, History, Arts, Marine biology, Freshwater ecology

## Abstract

Our study explores the use of Italian still-life paintings from the Early Modern Period (16^th^-18^th^ centuries) as historical records of past Mediterranean aquatic biodiversity. Following an environmental history approach, we analysed taxonomic composition in paintings, first examining geographic and temporal variations shaped by technical and socio-cultural influences. After consideration of these factors, we performed a detailed ecological interpretation of depicted taxa. Our findings reveal a shift from freshwater to marine resource use, driven by evolving fishing practices and technological advances. Socio-cultural elements, such as culinary traditions, religion and aesthetics also strongly shaped species representation. We discuss ecological interpretation of the representation of vulnerable and emblematic Mediterranean species in light of climate change, overexploitation and species biogeography. Our research highlights the powerful role of paintings in reconstructing past exploited ecosystems, offering a unique perspective for informing contemporary conservation efforts.

## Introduction

Understanding the historical dynamics of human-nature interactions is essential for interpreting how societies have shaped—and been shaped by—their environments. Beyond traditional scientific approaches, the field of environmental history incorporates past perspectives into modern-day frameworks to reconstruct these dynamics, using data from paleontological, archaeological, historical, ecological and sociological records alongside analytical tools^1–3^. Thus, a remarkable diversity of data sources, ranging from archives and archaeological remains, to non-traditional sources more typical of the humanities and social sciences, such as oral histories, have been used by researchers to uncover historical patterns of resource exploitation, environmental change and societal values^2^.

In the case of aquatic systems and in particular marine environments, long-term historical human-nature relationships have been explored using early travellers’ diaries, ship logbooks and naturalists’ illustrations, providing glimpses into past marine biodiversity and species distributions^4–6^. Researchers have principally focused on individual, highly exploited populations, and informed about the impact of climate change and overexploitation of marine resources using, for instance, catch records^7^, archaeological excavations^8^, fishermen’s stories^9^, photographs^10^, merchandise inventories^11^, public records^12^, restaurant menus^13^ and a compilation of multiple sources^14,15^. Insights gained from these historical records provide opportunities to learn about population abundances, biodiversity, spatial structure and ecological functioning in the past, and to conduct conservation-relevant research for present and future remediation and restoration (see refs. ^15–17^).

In this perspective, art has been suggested as a powerful tool for reinterpreting human-nature interactions^15,18,19^. Artistic depictions of aquatic animals appear across cultures worldwide, reflecting unique symbolic and ecological contexts. These diverse traditions provide valuable insights into human perceptions of aquatic life, illustrating the dynamic interplay between biodiversity, cultural values, and artistic expression across different periods and regions. We have focused our research on European aquatic environments, where only a few studies (listed hereafter) have explored the use of art. In the Mediterranean Sea, Guidetti and Micheli^20^ studied 23 Roman mosaics (from the 1^st^–5^th^ centuries CE) representing large groupers (*Epinephelus marginatus*) portrayed in shallow waters. Today however, only small-sized individuals are observed nearshore, and large groupers are restricted to deep waters^20^. This suggests that groupers may have had larger body sizes during Roman times and that they were fished nearshore at that time, whereas they are now classified as vulnerable by the IUCN Red List due to overfishing^21^. Another study focused on watercolour images painted by a Dutch fish trader who recorded occurrences of fisheries target species and fish consumption in a handwritten *Fish book* around the 16^th^ century^22^. These records and images were compared with published trawl survey data from the 20^th^ century, and indicate an almost complete disappearance of large body size species (rays and sharks, sturgeon, ling), confirmed by a drop in the average trophic level of Dutch fishery catches from 1950 onwards^22^.

More recently, a citizen science project reviewed 1676 paintings from the Netherlands (15^th^-20^th^ century) and identified 66 different portrayed marine and freshwater fish species^23^. Although the collaborative identification of these species appeared limited, some interesting trends were observed by the authors. For example, an increase in the proportion of Atlantic cod (*Gadus morhua*) was found in paintings from the 15^th^ to the 16^th^ century, which coincides with the discovery of the Newfoundland cod stock in 1500^24,25^. Similarly, a study of Early Modern paintings in Europe allowed to discriminate aquatic representations between Atlantic and Mediterranean regions in still-life paintings from the 16^th^ to the 18^th^ century^26^. The taxonomic composition of aquatic biodiversity as represented in the paintings was used to assess the temporal evolution of aquatic socio-ecosystems across the two regions. In accordance with most of the previously cited literature, an overall decrease of represented taxa, and particularly of freshwater and migratory species, is observable across centuries, in line with records from studies in archaeology, history and biology^26^. More importantly, the same study revealed a convergence between the origin of the paintings and the biogeographic area of the species, showing that the painted representations are reliable testimonies of which species the artists could observe in their environment and on the fishmongers’ stalls.

Art therefore seems to provide a significant, if as yet little recognised, source of ecological information about the past. These pictorial representations were produced before the development and widespread use of photography, which is now recognised as a valuable tool for revealing information about the ecological conditions of the recent past and present^27^. However, a general lack of interest in art as a source of empirical ecological data has persisted due to the non-existence of concomitant scientific observation, and it has been argued that artistic depiction of species can be compromised by the artist’s aesthetics and anthropocentric perspectives^28^. When subjected to multiple interpretations, art should nonetheless be treated as a valuable repository of past biodiversity data which can help to better understand periods when structured scientific records were not yet kept or are no longer readily available, enhancing our understanding of long-term change in ecosystems.

We investigated the depiction of aquatic biodiversity using a historical ecology approach to study Italian still-life paintings from the Early Modern Period (16^th^–18^th^ century C.E.), following Tribot et al.^26^. We focussed our research on the Mediterranean region, where historical ecology studies are scarce when compared to the Atlantic Ocean^22,23,29^. Aquatic ecosystems and especially socio-ecosystems comprising species of high commercial value have long been affected by anthropogenic and climatic pressures^30,31^. This is particularly the case in the Western region of the Mediterranean Sea, which has been strongly impacted by longstanding exploitation of resources^32^. Our study period coincides with the spread of artistic representation in a naturalistic style, and with the flourishing of painting schools across Europe, and most notably in Italy^33^. With increased opportunities for travel and the cultural development of major art centres, artists were able to construct influential relationships that promoted prolific production of paintings, often inspired by iconic artists such as Leonardo da Vinci, Michelangelo, and Raphael. Beginning in the 16^th^ century, the interest shown by early naturalists for accurate representations of animal or vegetal specimens also played a part in the development of naturalistic principles in the portrayal of organisms^34^. This association between art and the natural sciences lasted throughout the Early Modern Period and into the beginning of the 19^th^ century, which marks the end of our studied period.

Our aim is to expand the acquisition of relevant data and to demonstrate the pertinence of art and the potential contribution that its study may make towards a better understanding of human-nature interactions. We assume in this study that still-life paintings depicting aquatic life can serve as a valuable proxy for exploring past biodiversity in Mediterranean aquatic socio-ecosystems. By systematically analysing these artistic representations, we are able to identify changes in species composition over time, providing critical insights into the dynamics of human-nature interactions. Improved knowledge of the past biodiversity of Mediterranean ecosystems gained through the examination of artistic representations may thus help to mitigate climatic and anthropogenic impacts on present-day biodiversity, and so facilitate conservation-relevant restoration.

## Results

### Variations in the identified aquatic environments and habitats of represented taxa

**Fig. 1:**
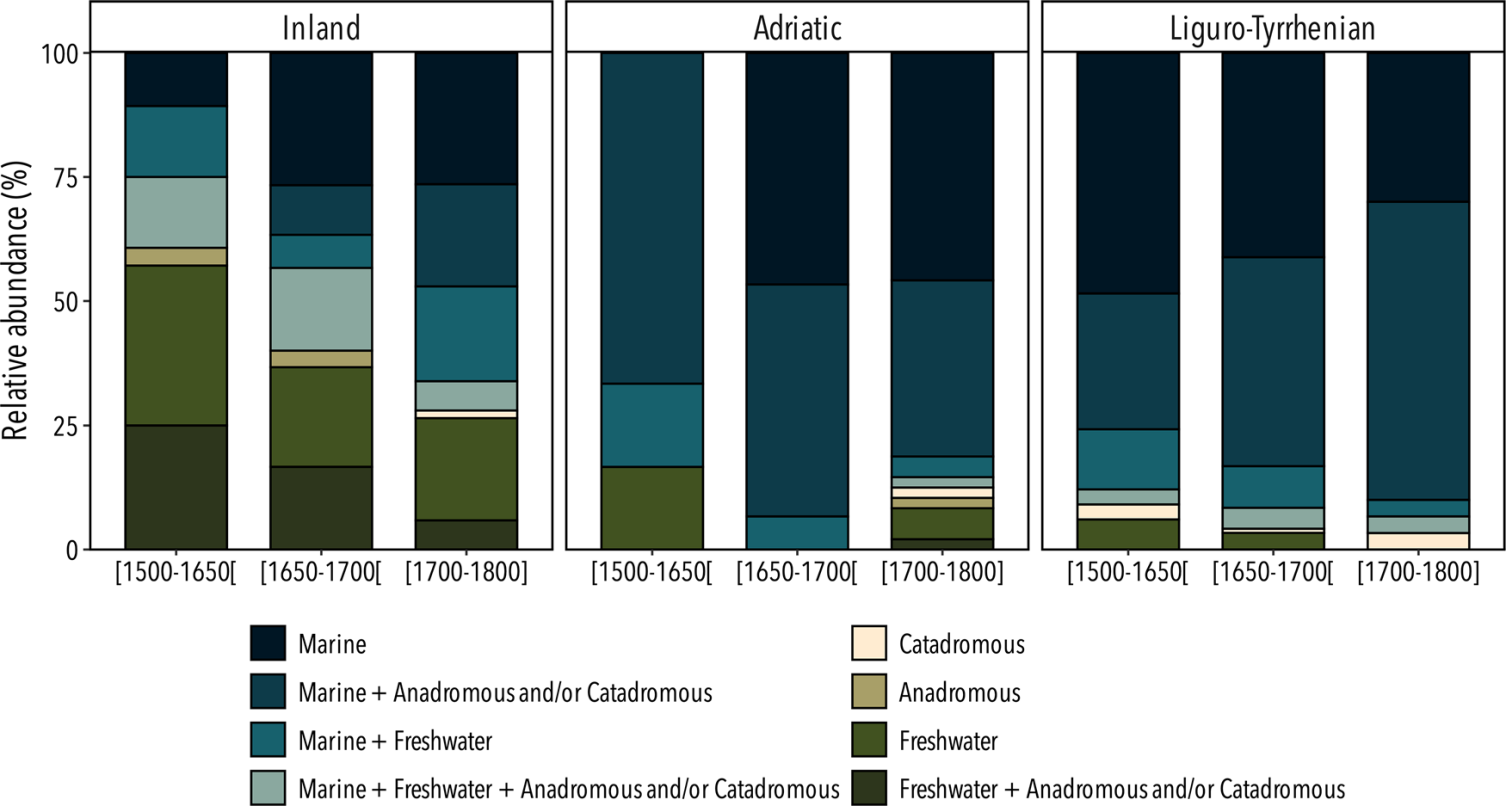
Frequencies of taxa from different environments represented in Italian paintings. Relative abundance (in %) of paintings representing taxa from freshwater, anadromous, catadromous, marine, and all other mentioned associations according to the three studied geographic zones (inland localities, Adriatic Sea, Liguro-Tyrrhenian Sea) and from three time-periods ([1500–1650] [1650–1700] [1700–1800]).

**Fig. 2:**
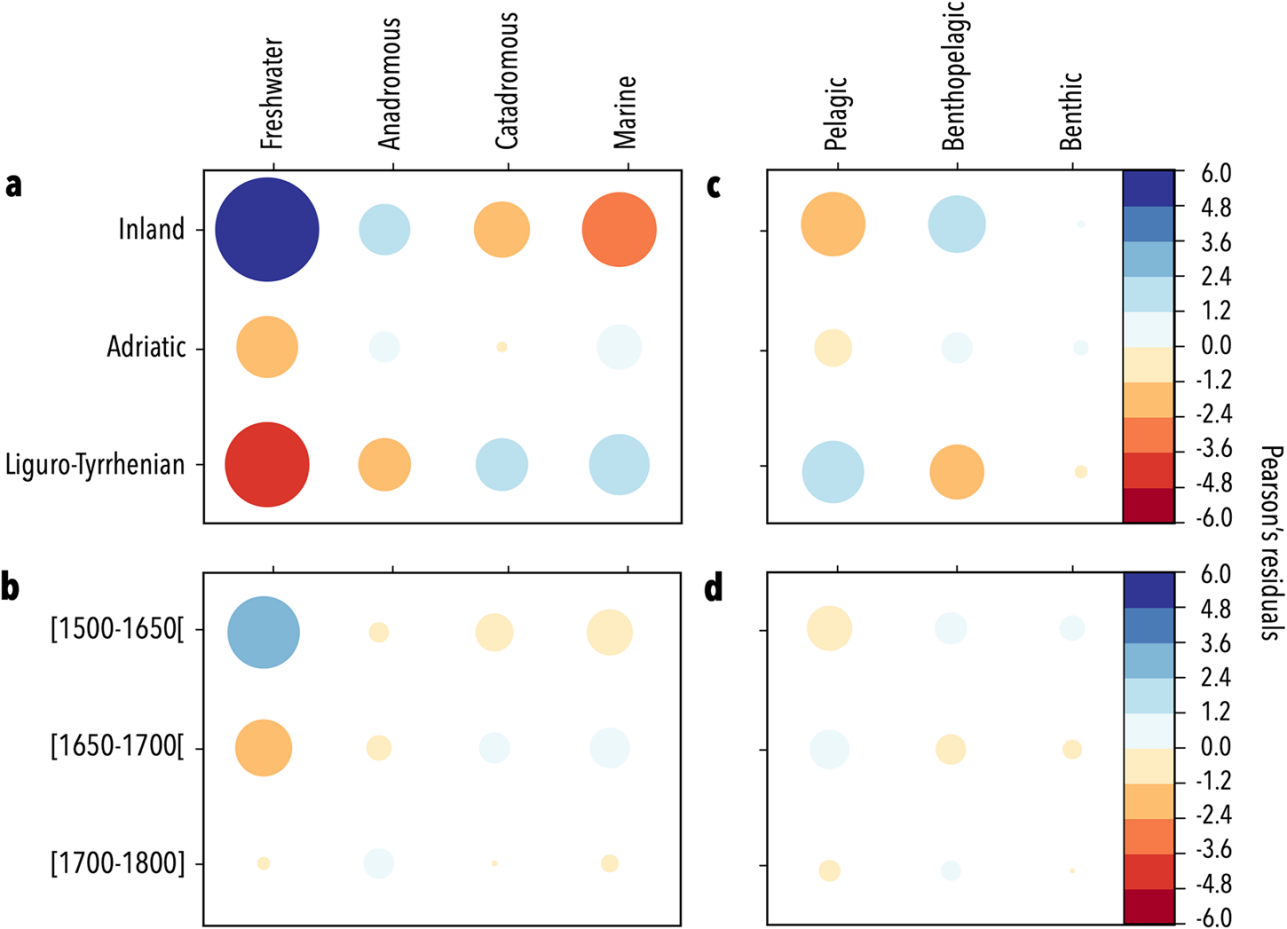
Pearson’s residuals of chi-squared tests for aquatic taxa across environments, habitats, geographic zones and time-periods. Pearson’s residual values of chi-squared tests performed on the proportion (%) of taxa from **a**, **b** aquatic environments (freshwater, anadromous, catadromous, marine) and **c**, **d** habitats (pelagic, benthopelagic, benthic) across **a**–**c** geographic zones (X-squared = 71.60, *p* value < 0.001 and X-squared = 13.89, *p* value = 0.008, for environments and habitats, respectively) and **b–d** time-periods (X-squared = 13.95, *p* value = 0.03 and X-squared = 2.88, *p* value = 0.58, for environments and habitats, respectively). Residual values are indicated by the coloured scale bars, with positive (blue) and negative (red) associations between variables, and dot size indicates the strength of the relationship.

All selected paintings depicted either animal displays, fishmonger stalls, fishing scenes, banquets or kitchens (see Fig. S1 for examples). All these paintings were classified under the style known as the still-life as they presented arrangements of inanimate aquatic organisms. Among the 92 identified taxa of Mediterranean origin, 71 were from marine environments, 14 were from freshwater and 7 were diadromous (4 anadromous and 3 catadromous). There was an overall dominance of the representation of marine organisms across spatiotemporal variables, representing from about 37% to 85% of total depictions (Fig. 1). However, painters from inland localities portrayed a higher proportion of freshwater organisms (up to 49%) compared to paintings from coastal areas (between 1% and 10%). Accordingly, a strong association of freshwater and anadromous taxa could be observed in inland paintings (Fig. 2a). Conversely, artists from coastal localities had a propensity for representing mostly marine organisms. For example, of the 14 paintings originating in the Adriatic for the period 1650–1700, all depicted at least one marine taxon (Fig. 1).

Over the studied period, the representation of freshwater organisms dropped dramatically, with a decrease in proportion by about half of represented freshwater taxa between the beginning and the end of the period in all zones (Fig. 1). This tendency could be observed statistically, as Pearson’s chi-squared results indicated a significant and positive association between paintings and freshwater organisms before 1650, while paintings executed after 1650 were negatively associated with these organisms (Fig. 2b).

We observed an overall dominance of benthopelagic organisms in paintings from inland and Adriatic locations, compared to paintings from the Liguro-Tyrrhenian coasts which were positively associated with pelagic taxa (Fig. 2c). The representation of habitats was not significantly correlated with time-periods, although an apparent increase in the representation of pelagic organisms could be observed in the 1650–1700 period (Fig. 2d). Similarly, the Pearson’s chi-squared tests across the three periods and geographic zones failed to show a significant relationship with trophic level (Chi-squared test, X-squared = 2.40, *p* value = 0.66), diet composition (X-squared = 6.50, *p* value = 0.89), or maximum size (X-squared = 2.07, *p* value = 0.72).

### Variations in taxa representation across geographic regions

The main depicted organisms were fishes (Actinopterygii), which represented about two thirds of total aquatic organisms (62–65%). Other portrayed classes were Malacostraca (i.e. crustaceans), Bivalvia (i.e. molluscs with two shells), Elasmobranchii (i.e. sharks and rays), Cephalopoda (i.e. octopus, squids and cuttlefish), Reptilia (i.e. marine turtles), Echinoidea (i.e. sea urchins and starfish), Gastropoda (i.e. marine snails), Mammalia (i.e. marine mammals), Octocorallia (i.e. corals) and Petromyzonti (i.e. lampreys). Although the present study only addressed paintings depicting aquatic organisms, we also encountered illustrations of terrestrial taxa in the inventoried paintings, such as tortoises, lizards, snakes, insects, and several recognisable plant species.

**Fig. 3:**
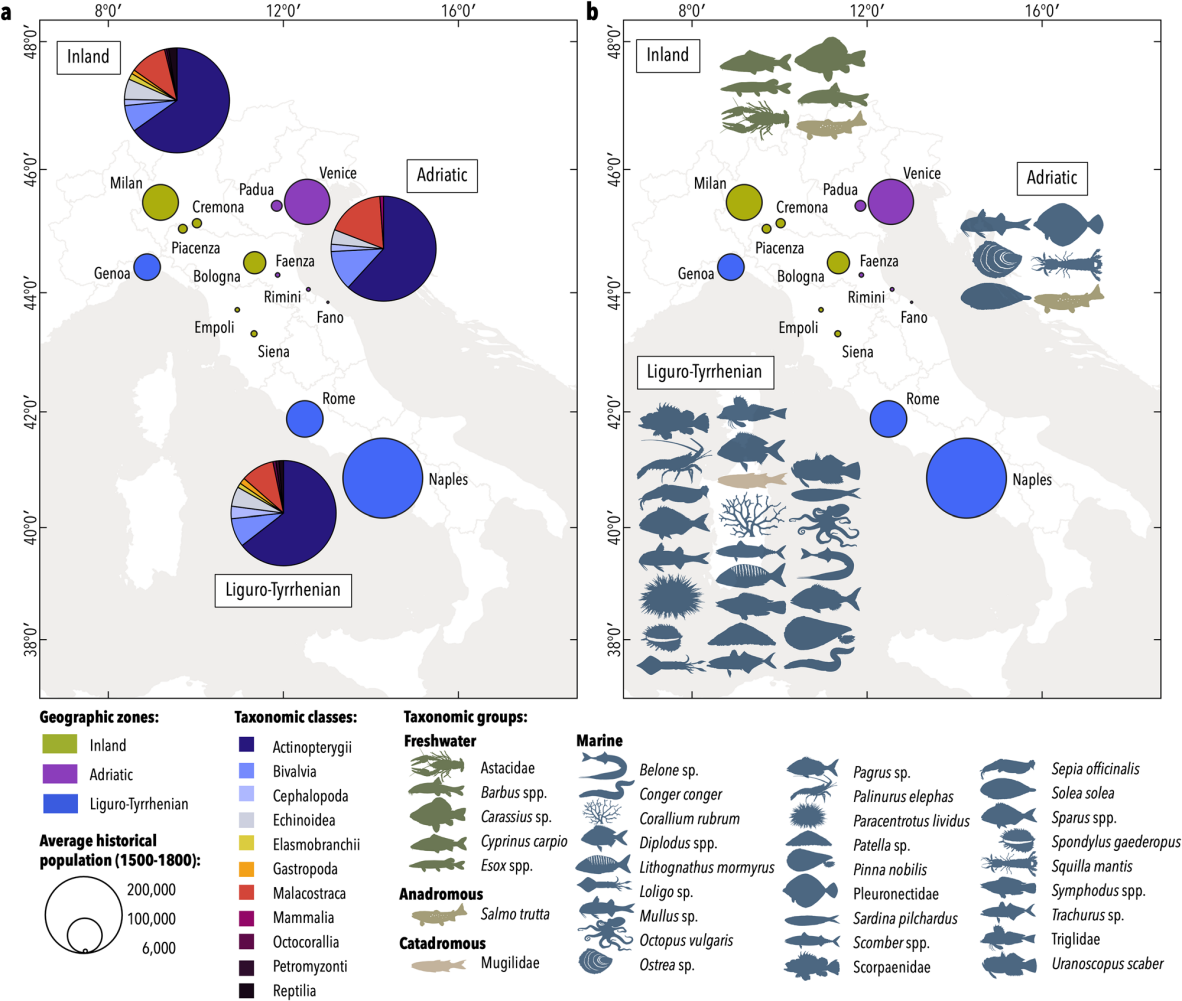
Taxonomic classes distribution and indicator species across geographic zones. **a** Distribution of the taxonomic classes represented in the paintings across the studied cities as grouped by geographic zones (inland localities, Adriatic Sea, Liguro-Tyrrhenian Sea) and **b** Indicator taxa identified in each geographic region by the Indicator Species Analysis, only taxa significantly associated to the geographic zones are displayed (Table S1). The average historical population of cities is given in Fig. S2a.

The proportions of taxonomic classes were seen to vary across the different geographic zones (Fig. 3a). In the Adriatic, crustaceans and molluscs represented a higher proportion (17% and 12%, respectively) compared to the two other zones, where they were present in about 11% of paintings for crustaceans and 8% for molluscs. Artists from the Liguro-Tyrrhenian coasts depicted an overall higher diversity of taxonomic classes, with painted specimens of marine snails, corals, lampreys and marine turtles (Cheloniidae), which were absent from Adriatic Sea paintings. Only one painting originating from the Adriatic coast represented an aquatic mammal of Mediterranean origin (Eurasian otter, *Lutra lutra*).

We identified a total of 85 taxa in the paintings that originated inland, 56 taxa from the Adriatic and 104 taxa from Liguro-Tyrrhenian localities. In the inland paintings, the most represented taxa were freshwater fishes, mostly composed of common carp (*Cyprinus carpio*) and pike (*Esox* spp.), that were present in 65 of the 126 paintings. About 68% of the illustrated pikes were identified at species level. The endemic species *E. cisalpinus* represented a roughly 55% occurrence while the northern pike *E. lucius* was found in fewer than 13% of paintings depicting pikes. Other commercially important taxa, such as crayfish (Astacidae), crucian carp (*Carassius* sp.), barbel (*Barbus* spp.) and the two anadromous taxa, sturgeon (*Acipenser* spp.) and trout (*Salmo trutta*), were also frequently depicted by inland painters (Table 1). In the case of barbels, about 24% were identified as *B. plebejus*, 19% as *B. tyberinus sensus lato*, 14% as *B. fucini*, and 43% could not be identified.

**Table 1:** Frequency of occurrences (in %) of taxa identified in the corpus of paintings from the Early Modern Period in Italy, according to the geographic zones where the paintings were located (inland localities, Adriatic Sea, Liguro-Tyrrhenian Sea)

Taxa	Inland	Adriatic	Liguro- Tyrrhenian
**Freshwater**			
*Abramis brama*	4.7	1.5	1.1
Astacidae	15.7	3.1	4.3
*Barbus* spp.	10.2	3.1	2.7
*Carassius* sp.	3.9		
Coregoninae	1.6		
*Cyprinus carpio*	35.4	3.1	6.5
*Esox* spp.	29.9	9.2	5.4
*Lutra lutra*		1.5	
*Perca fluviatilis*	3.1	1.5	0.5
*Potamon fluviatile*	0.8	1.5	1.6
*Squalius cephalus*	1.6		
*Tinca tinca*	5.5	1.5	2.7
**Anadromous**			
*Acipenser* spp.	5.5	7.7	3.2
*Alosa* sp.	3.1	1.5	3.8
*Petromyzon marinus*	0.8		1.1
*Salmo trutta*	4.7	3.1	0.5
**Catadromous**			
*Anguilla anguilla*	15.0	9.2	9.7
*Dicentrarchus labrax*	10.2	10.8	16.2
Mugilidae	13.5	20.0	33.9
**Marine**			
*Belone* sp.	5.5		10.8
*Boops boops*			2.2
Brachyura	4.7	6.2	2.7
Cardiidae		1.5	1.1
Caridea	0.8	1.5	0.5
*Cheilopogon heterurus*		1.5	
Cheloniidae	4.7		3.8
*Conger conger*	1.6		5.9
*Corallium rubrum*	0.8		9.7
*Diplodus* spp.	2.4	1.5	13.5
*Engraulis* sp.	4.7	6.2	1.6
*Ensis siliqua*	1.6	6.2	5.4
*Epinephelus* spp.	1.6		
*Homarus gammarus*	17.3	12.3	8.1
*Lithognathus mormyrus*			7.6
*Loligo* sp.	3.9	1.5	17.3
*Lophius* sp.	2.4		3.8
*Maja* sp.	16.5	12.3	10.3
*Merluccius merluccius*	3.9	3.1	8.1
*Mullus* sp.	17.3	36.9	43.8
*Muraena helena*	1.6		4.9
*Mytilus* sp.	3.1	3.1	1.6
*Octopus vulgaris*	1.6		7.0
*Ostrea* sp.	13.4	35.4	11.4
*Pagellus* sp.	4.7	1.5	10.8
*Pagrus* sp.			2.7
*Palinurus elephas*	11.0	1.5	29.7
*Paracentrotus lividus*	3.9		17.3
*Patella* sp.	0.8		8.1
*Pecten maximus*	6.3	6.2	2.7
*Pinna nobilis*	0.8		4.9
Pleuronectidae	3.1	15.4	5.4
*Sardina pilchardus*	7.1	4.6	10.3
*Sarpa salpa*	1.6	3.1	9.2
*Sarda sarda*		1.5	2.7
*Scomber* spp.		4.6	11.4
Scorpaenidae	9.4	1.5	33.5
*Sebastes* sp.	2.4	1.5	2.7
Selachii	3.1	4.6	2.7
*Sepia officinalis*		1.5	14.1
*Serranus scriba*	0.8		2.2
*Solea solea*		16.9	4.9
*Sparus* spp.	5.5	4.6	22.7
*Sphyraena* sp.			2.7
*Spondylus gaederopus*	0.8		12.4
*Squilla mantis*	2.4	10.8	3.2
*Symphodus* spp.	0.8		8.1
Scombridae	0.8		3.2
*Torpedo torpedo*			1.6
*Trachinus* spp.	0.8	1.5	2.2
*Trachurus* sp.	0.8	1.5	9.2
Triglidae	16.5	18.5	36.2
*Umbrina cirrosa*		1.5	0.5
*Uranoscopus scaber*	1.6		8.1
Veneridae	1.6	4.6	0.5
*Xiphas gladius*			2.7
*Zeus faber*	7.1	3.1	4.3

Only taxa with occurrences above 1% are displayed.

**Fig. 4:**
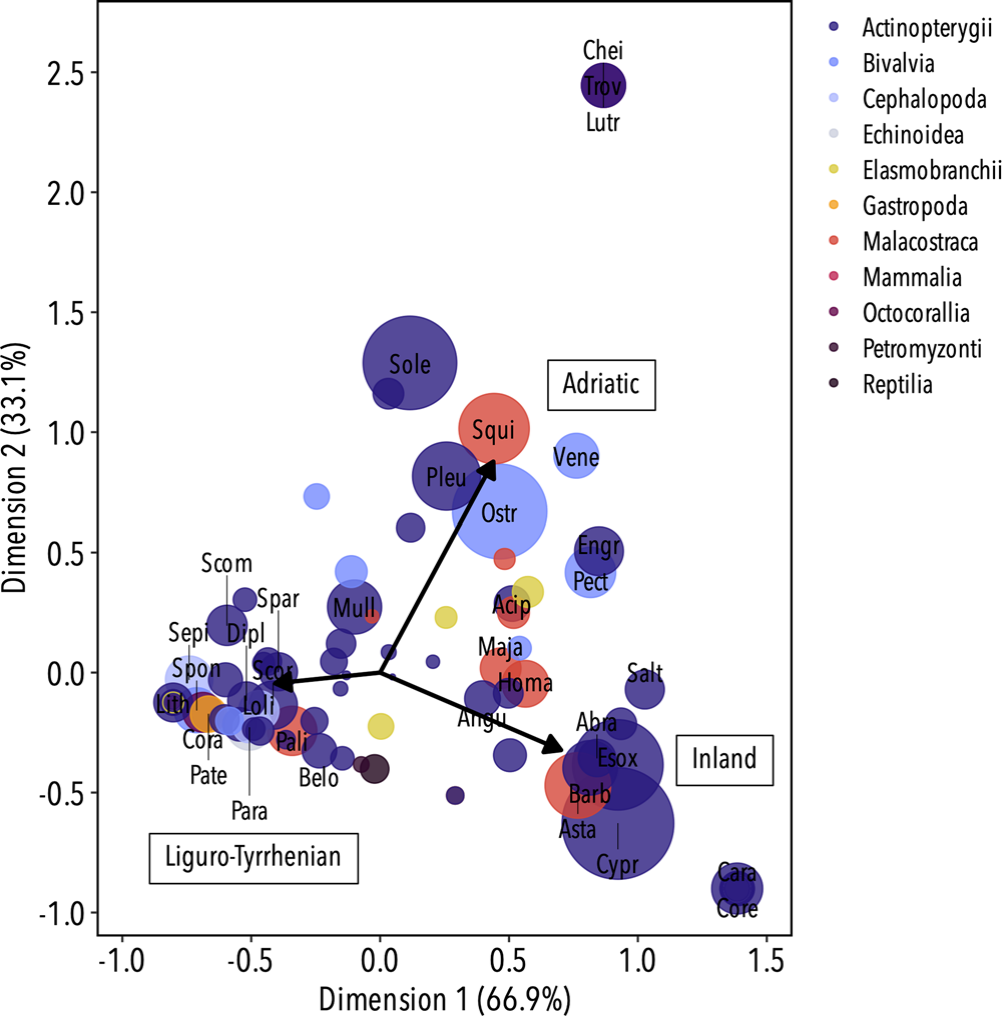
Taxa diversity in relation to geographic zones. Correspondence Analysis (CA) map of the taxa diversity represented in paintings from the Early Modern Period in relation to the three geographic zones (inland localities, Adriatic Sea, Liguro-Tyrrhenian Sea). The size of the dots corresponds to the contribution to the CA ordination. Only taxa with a cumulative contribution of 80% are labelled. Cypr, *Cyprinus carpio*; Sole, *Solea solea*; Ostr, *Ostrea* sp.; Esox, *Esox* spp.; Pleu, Pleuronectidae; Asta, Astacidae; Squi, *Squilla mantis*; Mull, *Mullus* sp.; Barb, *Barbus* spp.; Scor, Scorpaenidae; Cara, *Carassius* sp.; Vene, Veneridae; Homa, *Homarus gammarus*; Pali, *Palinurus elephas*; Chei, *Cheilopogon heterurus*; Lutr, *Lutra lutra*; Sepi, *Sepia officinalis*; Spon, *Spondylus gaederopus*; Para, *Paracentrotus lividus*; Spar, *Sparus* spp.; Engr, *Engraulis* sp.; Scom, *Scomber* spp.; Salt, *Salmo trutta*; Maja, *Maja* sp.; Cora, *Corallium rubrum*; Loli, *Loligo* sp.; Acip, *Acipenser* spp.; Lith, *Lithognathus mormyrus*; Abra, *Abramis brama*; Pect, *Pecten maximus*; Angu, *Anguilla anguilla*; Dipl, *Diplodus* spp.; Pate, *Patella* sp.; Ensi, *Ensis siliqua*.

Paintings from coastal areas depicted a wider diversity of marine organisms, with the most represented fishes being red mullet (*Mullus* sp.), grey mullet (Mugilidae) and gurnard (Triglidae) in both the Adriatic and the Liguro-Tyrrhenian Sea (Table 1). However, there was a geographic difference in the representation of marine taxa across both coastal areas as observed in the Correspondence Analysis (Fig. 4). Painters from the Adriatic depicted fewer taxa, consisting mainly of representations of oysters (*Ostrea* sp.) and flatfish (common sole (*Solea solea*), flounder (Pleuronectidae)), together with shellfish (i.e. crustaceans and molluscs) such as the pod razor (*Ensis siliqua*) and Venus clam (Veneridae). Liguro-Tyrrhenian artists represented a larger diversity of marine organisms, with scorpionfish (Scorpaenidae), spiny lobster (*Palinurus elephas*), seabream (*Sparus* spp. and *Diplodus* spp.), squid (*Loligo* sp.), sea urchin (*Paracentrotus lividus*), and cuttlefish (*Sepia officinalis*) being some of the most represented taxa (Table 1).

Of the 92 Mediterranean taxa identified in the paintings, we determined 35 indicator taxa within spatial boundaries, most of which corresponded to the taxa identified by the multivariate statistical technique (Fig. 4). Five freshwater taxa and one anadromous fish species were strongly and significantly associated with paintings from inland localities, while only marine taxa (except for trout and grey mullet) were considered good indicators of Adriatic and Liguro-Tyrrhenian paintings (Fig. 3b). Only six taxa were associated with the Adriatic, while up to 24 taxa were significantly related to Liguro-Tyrrhenian paintings, with red mullet associated with both localities.

### Variations in taxa representation across time-periods

Regarding time-periods, the most frequently represented freshwater taxa in still-life paintings from 1500–1650 were pike (25% of occurrence), common carp (25%), crayfish (12%), barbel (9%), bream (*Abramis brama*, 6%), and tench (*Tinca tinca*, 4%). The representation of most of these taxa significantly decreased during the following centuries (Table 2). Conversely, we observed an increase of several of the depicted marine organisms, notably cephalopods, during the period 1650–1700 compared to 1500–1650 (Table 2). Remarkably, some marine taxa were never found in paintings before 1650, for example, the conger (*Conger conger*), angler fish (*Lophius* sp.), moray (*Muraena helena*), rockfish (*Sebastes* sp.) and stargazer (*Uranoscopus scaber*).

**Table 2:**
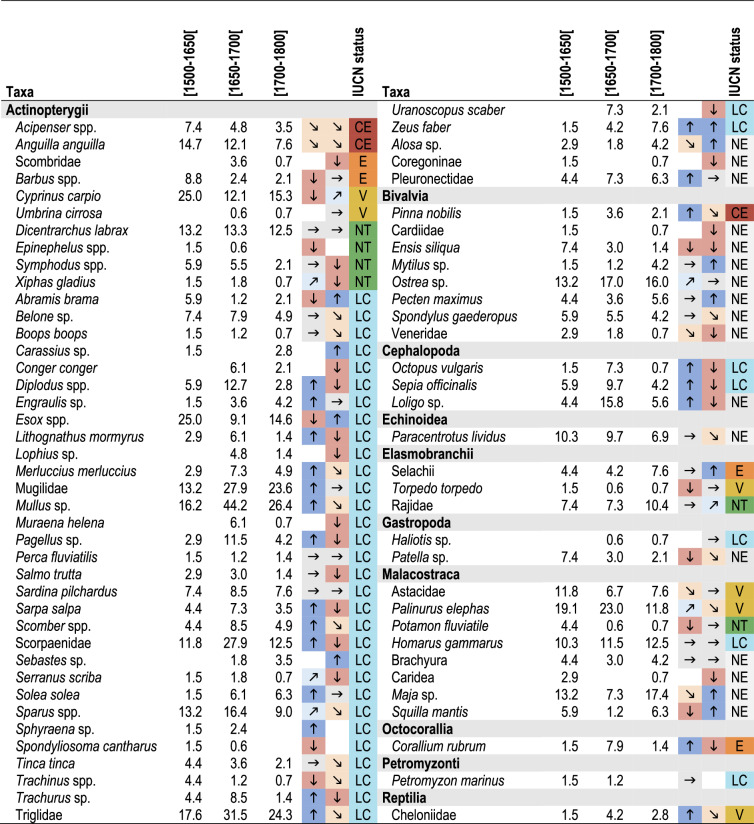
Frequency of occurrence (%) of the identified taxa from the corpus of Italian paintings from the Early Modern Period

Arrows represent increasing and decreasing trends in the frequency of occurrences of taxa across time-periods. Taxa were classified by their current IUCN status *CE* critically endangered, *E* endangered, *V* vulnerable, *NT* near threatened, *LC* least concern, *NE* not evaluated. Only taxa represented in at least two periods are displayed.

We observed no trends in the temporal variations of taxa sorted according to their current IUCN status (Chi-squared test, X-squared = 9.99, *p* value = 0.44). However, when we considered the taxa individually, we detected an overall decline in the representation of all taxa listed as critically endangered (CE), endangered (E) and vulnerable (VU), except for common carps and sharks (Selachii) (Table 2). The representation of emblematic Mediterranean taxa such as sturgeon, tuna (Scombridae), grouper (*Epinephelus* sp.), swordfish (*Xiphas gladius*), wrasse (*Symphodus* spp.), noble pen shell (*Pinna nobilis*) and red coral (*Corallium rubrum*) drastically decreased from the period 1650–1700 to 1700–1800 (Table 2).

**Fig. 5:**
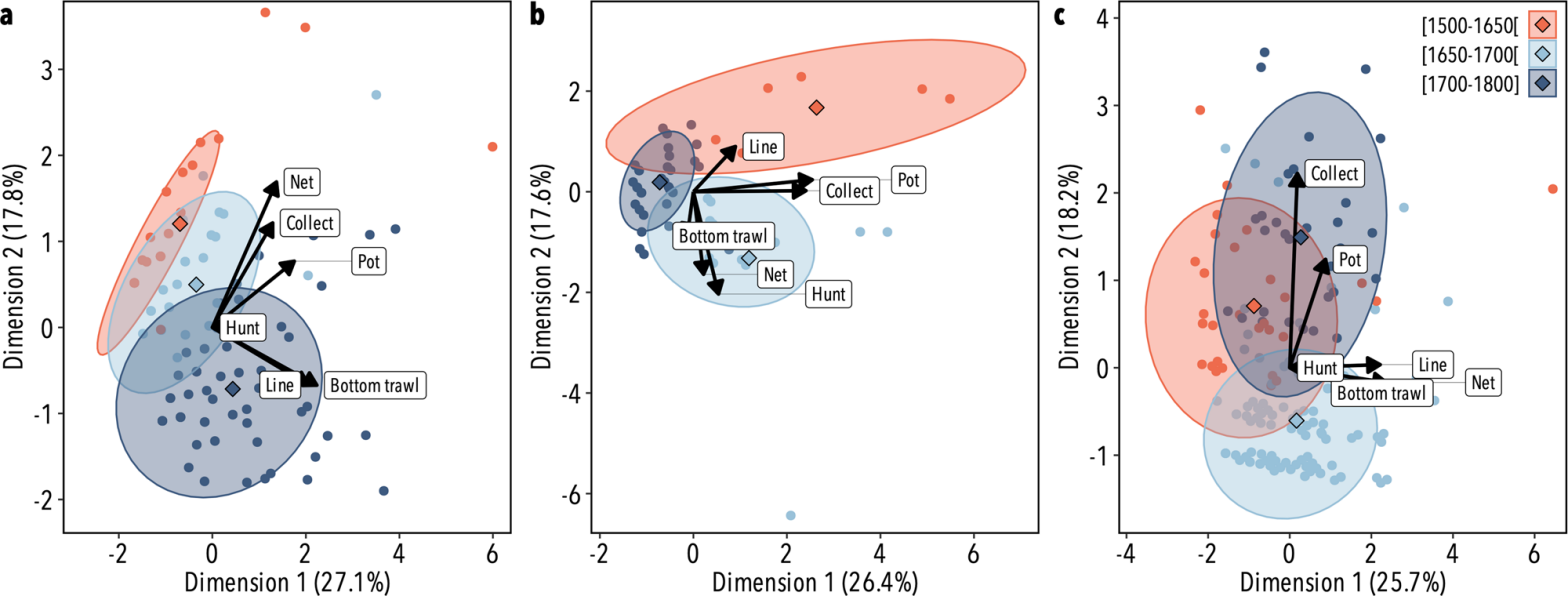
Taxa distribution according to fishing gear. Multiple Correspondence Analysis (MCA) based on the number of paintings in which taxa were identified according to fishing gear (bottom trawling, hand collection, hunting, line fishing, netting, fish pots) and grouped according to the studied time-periods in **a** Inland, **b** Adriatic and **c** Liguro-Tyrrhenian paintings. Percentages on the axes indicate percentages of inertia explained by each MCA dimension. Confidence ellipses are represented (90%) as well as the mean point of each time-period (diamonds).

In all geographic zones, the temporal variations in the representation of taxa appeared to be associated with the fishing gear employed to harvest or to fish these taxa. Despite an absence of statistical evidence for these variations (Chi-squared test, X-squared = 16.72, *p* value = 0.08) we did, however, observe a temporal transition from traditional fishing methods, such as the use of nets, pots or collection by hand in paintings from 1500–1650, towards more advanced technological fishing gear such as lines and bottom trawls in paintings from the end of the studied period (Fig. 5). This transition was notably observed in inland and Adriatic paintings, indicating an increase in depictions of taxa fished using bottom trawls from the period 1650–1700 (Fig. 5a, b).

## Discussion

We have explored the historical representation of aquatic resources in Italian still-life paintings as an indicator of past aquatic socio-ecosystems. In this study, still-life paintings were used as a selective and powerful lens on the human-nature relationship rather than as a comprehensive archive of aquatic biodiversity. Our focus was on paintings from the Early Modern Era, a period that remains understudied due to the lack of systematic scientific records, particularly for the Mediterranean Sea. Existing historical archives, when available, are often fragmented and country-specific, making access challenging – especially in Italy which only unified in 1861, leaving many historical documents dispersed and largely undigitized.

Using an environmental history approach, we analysed paintings from various Italian regions and periods, examining how artistic representations of aquatic organisms reflect human interactions with aquatic species. Our interpretation of aquatic ecosystems through still-life paintings is shaped by two key filters: a technical filter (fishing techniques, aquaculture and trade) and a socio-cultural filter (culinary preferences, aesthetic choices and symbolism), both of which are developed in the first two sections of our discussion. Taking these filters into account has enabled us to develop a more nuanced ecological interpretation of the variations in the depicted taxa, considering ecological factors such as biogeography, climate and habitat changes, resource overexploitation, and the presence of introduced or cryptic species.

Still-life depictions of aquatic species reflect the exploitation of aquatic resources and technological advances in fishing in Italy. In the Mediterranean Basin, aquatic organisms have long been a central resource for subsistence^35^. Before the 16^th^ century, early fisheries in Italy relied heavily on freshwater and anadromous species which were harvested traditionally from artificial ponds and brackish coastal lagoons, especially in the North^35^. On the other hand, maritime fishing remained a small-scale activity until the 16^th^ century, primarily aimed at fulfilling the subsistence needs of local communities^36^. The distinction between fishery activities of inland and coastal localities was clearly visible in the art, with inland paintings associated with freshwater and anadromous species, while marine organisms dominated coastal art. These observations correspond to the most consumed organisms at times when people relied principally on species available in their own locality^36^. For instance, shellfish were abundantly depicted in paintings from the Adriatic region. Bivalves such as razor pods and Venus clams were hand-harvested traditionally along the North Adriatic coast for centuries^37^. Similarly, oyster cultivation has long been practiced in the Adriatic, with remains dating back to Roman times^38^. This custom of farming, together with shellfish consumption, made these taxa prominent subjects in local paintings over the centuries.

The presence of marine organisms, and notably shellfish, was nonetheless extensive in inland paintings throughout the period, highlighting the scale of trade across the Italian peninsula. Trade was an essential part of early fisheries activities, and historical sources indicate prosperous exchanges from inland lakes and rivers towards coastal localities during the 14^th^–15^th^ centuries^39^. Traditional merchant routes such as the *via Aemilia* were later followed on a seasonal basis by coastal merchants to transport fresh marine fish to inland localities^39^. For instance, grey mullets were abundantly represented throughout the studied period in all regions. They were easy to catch in coastal and brackish waters due to their consistent presence and migratory routes^40^, and their high economic value explains the successful trade across regions and throughout centuries^41^. In the paintings we observed a noteworthy similarity between inland and Adriatic paintings in the depiction of marine organisms: this similarity reflects regional exchanges which were facilitated by the preservation of these marine organisms which could then more easily be transported across the Po River and along trading routes.

Prior to the 16^th^ century, fishing in the Mediterranean relied on traditional methods such as the use of hand nets, pots, and traps. These techniques, primarily used for fishing and harvesting freshwater species, coincided with recurrent representations of freshwater organisms in still-life paintings before 1650. During that time, the exploitation of marine resources was dominated by coastal lagoon fishing, and artists often depicted species caught inshore, such as grey mullets. Historical research highlights the prevalence of these local fishing practices in the Mediterranean until the 17^th^ century, when the economy began to shift toward coastal exploitation^39^. This shift was driven by new types of fishing gear, such as bottom trawls, longlines, drift nets, and gill nets which greatly increased the exploitation of marine resources^42^. As these methods were adopted, the diversity of catches expanded, which in turn influenced the diversity of species depicted by artists.

Trawling methods targeting benthic species emerged in the 15^th^ century and had expanded significantly by the 18^th^ century^43^. The rise of entire fleets dedicated to bottom trawling led to an increase in both the yield and diversity of catches^44,45^, which coincided with a growing depiction of benthic organisms in still-life paintings. This trend was particularly prominent in the Adriatic Sea, where benthic and benthopelagic species were widely represented from the 17^th^ century onward. The large, shallow continental shelf of the Northern Adriatic facilitated the expansion of trawling, making it common practice by the mid-17^th^ century^39^. Artists in the region, inspired by the rich variety found in fishermen’s catches and on fishmongers’ stalls, frequently depicted bottom-dwelling species such as flatfish, stargazers, John Dory (*Zeus faber*), anglerfish, in addition to benthopelagic sharks and rays. In contrast, the narrow continental shelf along the Liguro-Tyrrhenian coasts of Italy limited the use of trawling, resulting in fewer benthic fish appearing in paintings from this area.

The use of Sardinal drift nets and Tartane trawlers became widespread by the 17^th^ century in the quest for catching pelagic fish^43,46^. However, unlike benthic species, pelagic fish remained underrepresented in Italian still-life paintings, making up overall fewer than 17% of the organisms depicted in art. This trend contrasts with previous observations at the European scale, where painters from Northern Europe increasingly represented pelagic fish across the same studied period^26^. Two exceptions, however, concern the migratory pelagic tuna and the swordfish. The use of specific types of gear such as tuna madrague to target these large species was widespread by the 17^th^ century^47^, and is reflected in artistic representations from between 1650 and 1700, suggesting increases in tuna and swordfish catches. However, their depiction sharply declined in the 18^th^ century, likely reflecting a change in migratory routes towards the open sea^47,48^. In Italy in particular, tuna catches significantly dropped from the 17^th^ century onwards due to climatic change, and using traps ceased to be commercially viable^49^. Conversely, artistic representations of sharks increased over the studied period, likely due to their incidental capture as by-catch in longline fishing. This trend may also correlate with the growing use of commercial longline fishing from the 18^th^ century onward^43^.

Socio-cultural factors, including culinary preferences, aesthetic choices, and religious symbolism, also influence the depiction of aquatic resources in art. The representation of aquatic species in still-life paintings attests to the culinary value of these organisms and provides a window onto the tastes of Italian social classes. Most paintings were either commissioned by affluent families and were out of the reach of ordinary people. As a result, the depicted species consisted primarily of fresh, high-quality fish, with few representations of preserved fish (salted, smoked or dried), which lacked the visual appeal of fresh fish but which were more accessible to the lower classes^39^. Most paintings tended to depict high-value specimens and those appreciated for their culinary interest. For instance, despite their significance in the Mediterranean diet^43^, small pelagic fishes such as sardines (*Sardina pilchardus*) and anchovies (*Engraulis* sp.) appeared in fewer than 10% of the studied paintings. These species were considered low-quality and were sold cheaply to the lower social classes, and were sometimes even deemed unhealthy according to the medical theory of humors^39^. Similarly, European eels (*Anguilla anguilla*), though heavily fished from the Medieval period onward^50^, showed a decline in representation through the centuries, linked over time to their association with lower-class consumption^51^. Conversely, crabs (Brachyura), mantis shrimp (*Squilla mantis*), and lobsters (*Homarus gammarus*) were commonly consumed by all social classes^37^ and figure frequently in the corpus, especially in the paintings of the Adriatic region. These edible shellfish in particular were artistically displayed in restaurant windows in Venice, as is still the case nowadays. The nutritional and economical significance of the depicted organisms was not analysed in this study but could be proposed for future research.

As underlined by Tribot et al.^26^, aesthetic choices may also determine the species compositions of still-life paintings. Artists of the Early Modern Period appear to have chosen species based on aesthetic qualities such as colour and shape, without regard for their culinary value. For example, the Recco family of painters frequently included vibrantly coloured species such as gurnard, red mullet, scorpionfish, and seabream (*Pagellus* sp.), using bold reds to contrast with darker elements in their compositions. Since Antiquity, red has been linked to power and the sacred, and Italian painters, by saturating their works with red, highlighted the preciousness of the depicted marine animals. This emphasis on colour is exemplified in Giovanni Battista Recco’s “*Still-life with fish and oysters*” (1653) (Nationalmuseum Sweden), where bright colours enhance the visual impact of the painting. In our still-life corpus, the presence of red coloured species was observed in about 65% of paintings, and in up to 79% of paintings originating from Naples, although we were not able to quantify precisely the aesthetic interest for painters. In addition to colour, some species may have been depicted because of their unique and intriguing shapes. Examples include seahorses (*Hippocampus guttulatus*), flyingfish (*Cheilopogon heterurus*), sunfish (*Mola mola*), the red-spotted box crab (*Calappa* sp.), spiny starfish (*Marthasterias glacialis*), and the angelshark (*Squatina squatina*). These species added an element of curiosity and novelty to the paintings, showcasing the artist’s creative choices rather than reflecting common culinary practices.

Still-life paintings from the Early Modern Period also often incorporated religious themes, reflecting the cultural and symbolic significance of certain objects. One notable example is the use of red coral, a material valued in Mediterranean cultures for its alleged protective properties^52^. In 17^th^ century Italy, “coralline” boats actively fished for coral along the peninsula and around the surrounding islands^53^. However, by the late 18^th^ century, declining commercial demand had led to a regulated suspension of coral fishing, which further reduced harvests^53^. This shift is mirrored in art, particularly in paintings from the Tyrrhenian coast, where red coral appears in compositions featuring mythological figures, emphasizing its symbolic resonance.

Once technical and socio-cultural influences are considered, still-life paintings provide valuable ecological insights by reflecting geographic distribution of species, the effects of climate change and habitat modifications, the overexploitation of emblematic and endangered species, and the presence of introduced and cryptic species. Italian painters displayed a clear propensity for representing locally accessible taxa, which confirms a convergence between the origin of the paintings and the species’ biogeographic area, as demonstrated by Tribot et al.^26^. In freshwater environments, fisheries essentially targeted abundant nearshore benthopelagic fishes, such as pike, Eurasian perch (*Perca fluviatilis*), tench and common carp^36^, all widely depicted in paintings from inland localities, along with other commercially important freshwater taxa. These freshwater taxa were also abundantly represented in Flemish still-life works^26^, and Italian painters may have been influenced by these representations. Although the historical exploitation of these fishes coincides with still-life representations^36^, it is not possible to quantify the influence of cultural diffusion of paintings with the dataset at hand. The representation of anadromous taxa, such as sturgeon and trout, was also frequent in paintings originating from both the North of Italy and from the Adriatic coast, in keeping with fishery reports which described these species as abundant in the Po and Tiber rivers and in the upper Adriatic Sea until the 19^th^ century, but rare in the Liguro-Tyrrhenian Sea^54^.

Regional variations were also notable between the Adriatic and Liguro-Tyrrhenian coasts. Adriatic painters frequently included taxa from sandy habitats, such as common sole, flounder, mantis shrimp, pod razor and the Venus clam in their paintings, mirroring the dominance of sandy shores in the Northern Adriatic. For instance, when compared with Northern European representations^26^, the mantis shrimp was portrayed almost exclusively in Mediterranean paintings, and notably in the Adriatic, which corresponds to its natural biogeographical distribution. Liguro-Tyrrhenian paintings depicted a great diversity of taxa, including marine molluscs, crustaceans, corals, sea urchins and lampreys. This high diversity was likely due to the variety of habitats found on the Liguro-Tyrrhenian coast, such as sandy banks, *Posidonia* meadows and rocky habitats. According to historical sources, the variety and abundance of fishes, crustaceans, molluscs, cephalopods and sea urchins was nowhere higher than in fish markets in Naples (Central Tyrrhenian Sea), where they were artistically displayed on stalls^37,40^. Liguro-Tyrrhenian artists faithfully represented this diversity with scorpionfish, spiny lobster, seabream, squid, sea urchins, and thorny oysters (*Spondylus gaederopus*) figuring amongst the most represented taxa.

Climatic changes, particularly during the Little Ice Age, affected the geographic distribution and population dynamics of aquatic organisms. The cold, wet winters prior to the 16^th^ century were favourable for many freshwater species^55^, in particular for poikilotherm fishes. However, the warming trend after the 17^th^ century led to population declines. This tendency was observed in our study with the decrease of most freshwater species over time, corresponding to a continent-wide decline, as depicted in a recent study on still-life representations of aquatic organisms across Europe^26^. This decline was exacerbated by habitat changes due to human intervention. During the studied period and especially around the mid-16^th^ century, important habitat modifications occurred in inland lakes, rivers and coastal ponds of Europe, leading to the decline of many freshwater fish and invertebrate populations^56,57^. By the second half of the 17^th^ century, drainage of wetlands for the benefit of agriculture led to massive habitat loss, especially in Tuscany, Veneto and Rome^39^. For instance, the Italian population of sturgeon, a cold-adapted migratory species, began to fall in the 16^th^ century due to climatic changes and temperature rise, and then endured a further decline by the beginning of the 19^th^ century^54^ due to overfishing, habitat modification, and pollution. This trend is reflected after 1650 in a corresponding decrease in artistic representations of freshwater and anadromous taxa, such as sturgeon.

Many of the taxa identified in the corpus were commercially exploited for centuries along the Mediterranean coast. Today, several of these species are listed as vulnerable or endangered, and fisheries management plans have been established in recent decades to recover past populations^58^. However, there is a significant gap in scientific records pertaining to their ecological status during the 15^th^–18^th^ centuries. Indeed, most studies that have assessed the ecological condition of vulnerable species are time-based and cover either a distant past (i.e. are based on archaeological remains^8,59^) or the relatively recent centuries (i.e. are based on biological sources^47^ or fishery landings^48^). The presence and relative abundance of these species in still-life paintings provides insights into the timeline corresponding to the decline of these vulnerable species, since by the end of the studied period, painters were depicting many fewer of the taxa currently listed as critically endangered, endangered or vulnerable. An example of this is the portrayal of emblematic Mediterranean species such as marine turtles, noble pen shells and red coral, which all declined sharply in paintings made after 1650. Marine turtles have historically been exploited by fisheries for food and trade^60^, which is confirmed by their constant presence in still-life paintings throughout the period studied. They were, however, depicted more frequently between 1650–1700, likely due to the diversification of fishing methods and increased bycatch. The decrease in their representation towards the end of the 18^th^ century might reflect an early population decline, as was documented a century later in fishing records^60^. Similarly, red coral and the noble pen shell have been exploited since Antiquity in the Mediterranean Sea^52,61^. Both species were harvested particularly actively around the Italian peninsula and islands, notably in the south of Italy, culminating in a peak in exploitation during the 17^th^ century^53,62^. While the near exclusive presence of both species in the studied paintings from the Liguro-Tyrrhenian coast corresponds with their geographical range, the observed decrease in their representation after the year 1700 appears to reveal a global disinterest for these species and a potential population early decline.

The high level of detail in still-life paintings has allowed us to identify most species, although some cryptic and introduced species presented us with challenges. For example, Italian pikes are currently represented by two known species, the endemic cisalpine pike (*Esox cisalpinus*) and the northern pike (*E. lucius*), both of which are present in several Italian lakes and rivers^63^. The detailed representation of pikes in the corpus allowed species-level identification by experts for about two thirds of specimens, revealing about 80% cisalpine and 20% northern pike. This result indicates that the representation of northern pikes in Italian paintings might reflect a historical introduction of the northern species from outside the country, leading to introgressive hybridizations among Italian pike populations^64,65^. Moreover, the overall higher depiction of endemic pikes suggests that they used to be more abundantly available for fishermen than introduced pikes, yet today they are identified as vulnerable, affected by introgressive hybridization and competition with *E. lucius*^66^.

Conversely, some species such as the Italian barbels (*Barbus plebejus*, *B. fucini* and *B. tyberinus sensus lato*) were particularly difficult to differentiate in the paintings due to their similar morphological characteristics and frequent hybridization^67^. However, none of the allochthonous barbel that currently occur in Italy such as *B. barbus*, *B.cylcolepis* and *Luciobarbus graellsii*, were identified^68,69^. These species have generated introgressive hybridizations with endemic barbel species, leading to conservation issues and attesting their recent introductions^70–73^. The three species were identified in the paintings by experts, although some uncertainties remain. Similarly, we could not precisely differentiate between the Italian native crayfish species belonging to the genera *Austropotamobius*, nor was it possible to establish if some paintings depicted the allochthonous noble crayfish (*Astacus astacus*), which was historically introduced into Italian aquaculture in the 19^th^ century^74^. Earlier signs of its introduction were suggested in paintings, but more detailed research or direct close observation of the paintings by taxonomy experts is required.

Despite their historical significance, some commercially important species are scarcely represented in Italian still-life paintings. Groupers, for instance, were widely portrayed in Roman mosaics^20^ but are almost absent from Early Modern paintings, probably reflecting an early population decline. The torpedo ray (*Torpedo torpedo*) was frequently represented in ancient Greek and Roman art due to its various ‘medical’ applications^75^, but is similarly underrepresented in still-life paintings. Its declining use for medical purposes in the 4^th^ century likely explains this sporadic presence in Early Modern paintings. Similarly, Italian artists depicted very few marine mammals, unlike what can be found in Northern European paintings^26^. Only one walrus and two seals were found, all three in Arcimboldo’s *Water* (1586), which is a composite portrait combining aquatic creatures. However, neither of these species belongs to the Mediterranean fauna, as the portrayed seals lack resemblance with the only known Mediterranean species, the Mediterranean monk seal (*Monachus monachus*). This latter species, although abundant in ancient times, faced population collapse in the 16^th^ century due to human exploitation^76^, which may explain its absence in the paintings. Yet monk seals were also rarely depicted in early art, with the few known examples including Palaeolithic engravings from the Cosquer cave (27,000–18,500 BCE)^77^, ancient Greek coins (7–6^th^ century BCE)^78^ and a recently discovered Greek painted vase (6–5^th^ century BCE)^78^. The relative lack of more recent artistic representation may stem from the reputation of seals in myths and religious writings, where they were categorised as malignant animals among the sea monsters, a belief that stemmed since the spread of Christianity^78^.

In conclusion, the understanding of historical dynamics of human–nature interactions has proven to be challenging. On the one hand, while scientific monitoring has significantly shaped modern understandings of ecosystems, its scope is usually limited to recent decades, offering a relatively short temporal perspective that often overlooks pre-industrial ecological conditions^3^. On the other hand, more ancient historical archives are not easily accessible and, when available, quantitative information is only partial or imprecise^3^. These narrow views can inadvertently contribute to the shifting baseline syndrome described by Pauly (1995)^79^. In response to these limitations, researchers have increasingly turned to integrative and interdisciplinary approaches to reconstruct past environments and unravel long-term socio-ecological dynamics^1–3^. Building on an environmental history approach, our research emphasizes the value of art as a complementary source of ecological and historical data to explore long-term socio-ecological dynamics, especially for periods and places where scientific records are sparse or fragmented. In particular, still-life paintings from the Early Modern Period, renowned for their detailed representations of natural objects, serve as valuable visual archives for examining historical biodiversity. This study offers an analytical framework for interpreting these artworks as source of insight into past socio-economic dynamics, cultural practices, species distributions and ecological changes influenced by climate variability and human activity. At a time when aquatic ecosystems face unprecedented pressures from overexploitation, pollution and climate change, integrating artistic representations with scientific evidence provides a novel and interdisciplinary approach for enriching biodiversity research. By drawing from these diverse sources, we can develop more comprehensive conservation strategies rooted in long-term ecological awareness. Meanwhile, art offers a powerful medium for raising awareness about the urgent need to protect at-risk ecosystems, offering new pathways for engagement in biodiversity conservation.

## Methods

### Studied period: the early modern period

Historically, the Early Modern Period (16^th^–18^th^ century CE) can be defined as a time of societal transition, characterized by an early stage of modernity in which global expansion developed alongside technological advances. Directly following the Middle Ages, this period was marked in Europe by the cultural movement of the Renaissance, by the intellectual and philosophical Age of Enlightenment, and ended with two major events in the late 18^th^ century: the French Revolution and the Industrial Revolution^80^. Prior to the Early Modern Period was the longer climatic cooling event known as the Little Ice Age^81,82^. From the year 1400 CE onwards, a drastic drop in global temperatures gave rise to an agricultural crisis and the vast reorganization of land use. The gradual adaptation of Europeans to unpredictable and harsh climatic conditions resulted in fundamental societal changes^83^. Demographic expansion almost doubled during the 300-year period^84^ due to the intensification of land use and improvements in welfare, causing a greater demand for food and an expanding need for resources.

Fishing communities were particularly transformed during the Early Modern Period. The traditional harvesting methods predominantly used across the Mediterranean Sea were gradually replaced by larger commercial fishing activities in artificial ponds and at sea^43^. Simultaneously, an increase in the demand for fish in markets led to large-scale fishing campaigns in an attempt to increase both catch yields and species diversity^44,45^. In addition, overseas trade and transportation of aquatic organisms across Europe was made possible by the development of techniques of preservation and allowed fishmonger stalls to propose a wider diversity of species from various origins^40^. From the 17^th^ century, merchant routes such as the *via Aemilia* in Italy became major trading axes for freshwater and marine fish deliveries from inland localities to the coasts and vice versa^39^.

### Still-life: a realistic representation of nature

With such abundant product diversity, food items soon became symbols of wealth and economic growth^85^. This opulence rapidly became a source of inspiration for artists, who began to depict the natural and human world in a ‘realistic’ or ‘naturalistic’ manner^34^. From 1400 CE on, Dutch painters became inspired by flourishing international trade and extensively produced paintings representing inanimate objects (*stilleven*) and banquets (*banketje*)^86^. Still-life emerged, representing a broad range of representations, including dead or nearly dead animals in various contexts such as stalls, meals, kitchens, fishing. Empirical observation of food items led to their faithful reproduction on canvas, which would remain the major medium in figurative art until the invention of photography^33^. The development of still-life painting throughout Europe began in the 16^th^ century, encouraged by the interest that early naturalists^34^ displayed for accurate representations of living specimens. In Italy, the still-life (*cose naturali*) movement, influenced by early Flemish artists, became established first in Liguria, Veneto and Lombardy, where painters gathered in local schools^34^. During the 17^th^ and 18^th^ centuries, the development of the Neapolitan school of still-life painting, under the influence of Roman, Venetian and Netherlandish artists, established Naples as a major centre of cultural advancement^34^. For instance, the Recco family were Neapolitan artists active from 1618 to around 1800 who became known for their remarkable skill in depicting marine fauna^87^. Members of the Recco family painted an impressive number of still-life oil paintings representing aquatic organisms, some of which are exposed in art galleries and museums across Europe. The still-life remained the main representative genre until the end of the Early Modern Period, when modern technology allowed artists to explore other materials and techniques. This led to the movements of Romanticism and early Impressionism that began at the end of the 18^th^ century, and which saw artists exploring a distorted objective reality through the prism of their feelings, losing the realism and scientific accuracy hitherto present in artistic representations of objects.

### Data sources and treatment

Our study corpus of still-life paintings was constituted by contacting art museums, and by searching in online catalogues (Jocondes, AGORHA, Fondazione Zeri), auction databases, and national archives. Overall, we collected 384 paintings executed between 1515 and 1800 by 60 artists active in Italy (Table S1). In cases where the exact date of a painting was not indicated but where a time range was available, we used an average value to calculate the date of the painting. If a time range was not available, we determined the average date of the painting based on the median + 20 of the painters’ birth and death dates, assuming an artist to be active from their twenties to their death.

**Fig. 6:**
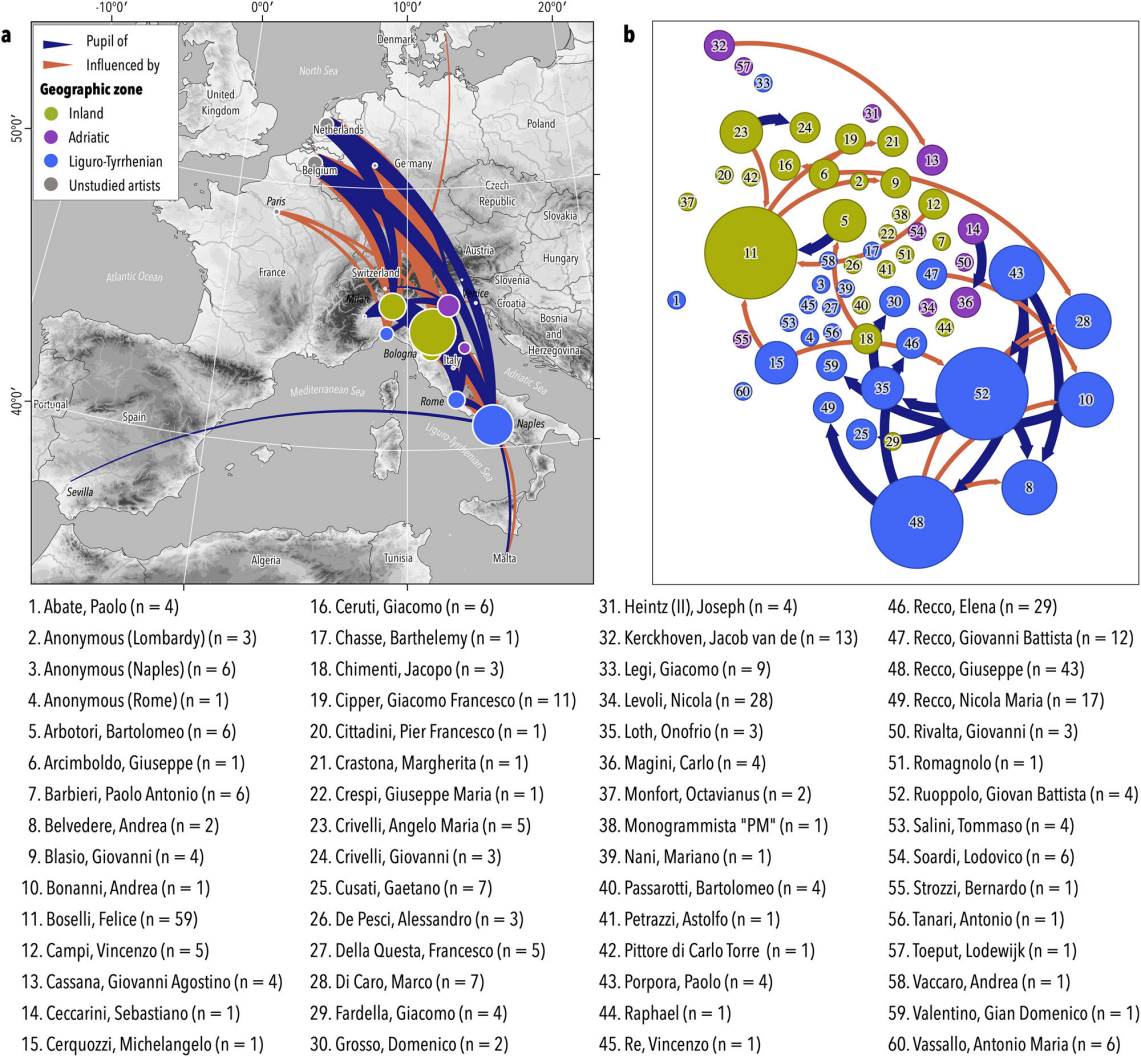
European painters’ relationships. **a** Cities of birth of the studied artists, unstudied teachers/pupils, and their influences on the studied painters, and **b** connexions among the studied artists. The sizes of the circles correspond to the degree of connexion among artists. See Table S2 for painters’ information. The numbers (*n*) next to the artists’ names refer to how many of their paintings feature aquatic organisms. Source: topography data from SRTM (http://srtm.csi.cgiar.org).

Information concerning the painter (cities of birth and death, major city of activity) and affiliations (masters/pupils, influences) were documented using the website of the Netherlands Institute for Art History (https://rkd.nl/en/explore/artists, accessed in May 2024) and other refs. ^88,89^ (Fig. 6). The major city of activity was used as the sampling locality but due to heterogeneity in the number of paintings across cities (Fig. S2), we grouped the cities according to their geographic location. This allowed us to link each painting to one of three geographic zones: inland localities (North of Italy), the Adriatic Sea, and the Liguro-Tyrrhenian Sea (Table 3). Inland localities were characterised by their proximity to freshwater rivers, ponds and lakes, notably the Po River Basin, and corresponded to cities with no nearby access to the sea. The two other localities (Adriatic and Liguro-Tyrrhenian seas) involved marine harbours and coastal cities situated less than 50 km from the sea (Fig. S2a). Adriatic localities were usually situated near coastal lagoons, such as Venice in the Veneto region and lowland coastal areas of the Po Basin. Paintings from the Ligurian and Tyrrhenian seas were grouped due to insufficient sampling size for Ligurian localities (*n* = 19 for all time periods). Preliminary analyses confirmed a similarity between paintings from these two areas, which supported a grouping of the artworks. We distinguished between paintings from the Adriatic and Liguro-Tyrrhenian seas due to the geographic differences of the two areas, as the Adriatic presents a large and shallow continental shelf compared to the narrower to nearly absent continental margin on the Liguro-Tyrrhenian side. Preliminary results allowed to divide the paintings into three time periods: [1500–1650] [1650–1700] and [1700–1800] (Table 3).

**Table 3:** Number of paintings collected in our corpus representing aquatic biodiversity and painted in Italy according to the defined time periods and geographic zones where the paintings were sourced

Geographic zone	[1500–1650]	[1650–1700]	[1700–1800]	Total
Inland (North of Italy)	29	31	70	**130**
Adriatic Sea	6	14	46	**66**
Liguro-Tyrrhenian Sea	34	120	34	**188**
*Total*	**69**	**165**	**150**	**384**

Bold values correspond to the sum of columns and lines.

Aquatic biodiversity (marine and freshwater) was assessed by the visual identification of specimens represented in the paintings, as described in Box 1. The Mediterranean Basin is a biodiversity hotspot, and several identified species represented in the paintings are endemic. For example, expertise from zoologists allowed to determine to the species level some newly described or revalidated species. This is the case for some freshwater fishes such as the cisalpine pike (*Esox cisalpinus*) which is endemic to the Adriatic Basin and diagnosable by its skin coloration pattern^63,90^, or for some Italian barbel species (*Barbus plebejus*, *B. fucini* and *B. tyberinus sensus lato* - including *B. samniticus*, that is indeterminable in the paintings). These fishes are endemic to the Padano-Venetian, Apulia-Campania and Tuscany-Latium districts respectively, and are distinguishable by their fin colour and by the presence/absence of a lobe on the lower jaw^67,91,92^, although some uncertainty remains (see Fig. S3 for examples). Conversely, some other cryptic species such as crayfish of the genera *Austropotamobius* could be neither determined nor discriminated from the potentially allochthonous Astacidae. Two exotic species, pufferfish (Tetraodontidae) and dragonfish (*Eurypegasus draconis*) were also identified, as were nine taxa of Atlantic Ocean/North Sea origin: Atlantic salmon (*Salmo salar*), Atlantic herring (*Clupea harengus*), Atlantic cod (*Gadus morhua*), lumpfish (*Cyclopterus lumpus*), haddock (*Melanogrammus aeglefinus*), pollock (*Pollachius* sp.), edible crab (*Cancer pagurus*), seals (Phocidae) and walrus (*Odobenus rosmarus*). The presence of Atlantic Ocean/North Sea taxa is not surprising, given that Italian artists used to travel around Europe, notably to Belgium and the Netherlands, where they were either inspired by local fishmonger displays or influenced by other artists (Fig. 6). Because these taxa are allochthonous to the Mediterranean Sea, they were not included in the analyses.

Every taxon was characterized by its environment (freshwater, anadromous (adults migrate into freshwater to spawn), catadromous (adults migrate into salt water to spawn), marine) and habitat (pelagic, benthopelagic, benthic) using FishBase (https://fishbase.mnhn.fr) for fishes and SeaLifeBase for other groups (https://www.sealifebase.ca) (see Table S3). Furthermore, when data were available, trophic level, diet composition, maximum size and IUCN status were determined. Fishing gear and methods used to collect organisms during the studied period (bottom trawling, hand collection, hunting, line fishing, netting, fish pots) were also identified following the authors’ expertise.

To assess the potential use of paintings as a proxy of past aquatic biodiversity, we first examined the taxonomic richness (S) using randomized accumulation curves of the ‘vegan’ R package^93^, considering the number of paintings as a measurement of sampling effort. By randomising the taxa observations, interpolated accumulation curves allowed to perform extrapolation for incomplete sampling and to identify the minimum number of paintings to sample in order to detect at least 95% of taxonomic richness, denoting an exhaustive inventory. The comprehensiveness and quality of the corpus were assessed as explained in Box 2.

Spatiotemporal trends in the representation of aquatic taxa were first addressed by analysing the frequency of occurrences (%) of the representation of taxa from each environment (freshwater, anadromous, catadromous, marine) and habitat (pelagic, benthopelagic, benthic) within the corpus according to geographic zones and time-periods. Residuals from Pearson’s Chi-square analyses (*chisq.test* function from the ‘stats’ package) were plotted to determine if the representation of taxa from specific environments and habitats was dependent on spatiotemporal variations. Similar analyses were performed on the trophic level, diet composition, maximum size and IUCN status of all taxa.

To assess potential differences in the representation of taxa in the paintings from the different spatiotemporal groups, the relative frequency of occurrence (%) for taxonomic classes and individual taxa was calculated for each geographic zone. We used a Correspondence Analysis (*CA* function, ‘FactoMineR’ package) based on a contingency table to visually interpret the associations between specific taxa and geographic regions. We then determined indicator taxa using the relationship between the occurrence values and the paintings based on the ‘indicspecies’ package. Temporal variations in the representation of specific variables such as fishing gear were addressed using a Multiple Correspondence Analysis (*MCA* function) based on the number of taxa identified across paintings within the studied time-periods. The frequency of occurrence (%) of taxa was then plotted to identify the taxa with the most variation across time-periods.

All statistical analyses were performed using R software^94^.

Box 1 Identification of taxa Aquatic organisms were identified at the lowest taxonomic level by the authors and experts (see ‘Acknowledgements’) following the INPN taxonomic ref. ^95^. Of the 130 identified organisms, 86 were determined at the species level, 27 at the genus level and 17 at the family level (Table S3). Similar morphological species (e.g. species from the Triglidae or Pleuronectidae families) were grouped for statistical representativeness, resulting in 92 taxonomic groups referred to as taxa. Each painting was analysed based on the presence or absence of taxa rather than specimen count (see Box Fig. 1), because the latter was considered to be a subjective value, influenced by the painter’s style and the aesthetics of the painting.

Of the 3641 aquatic specimens accounted for in the corpus, 15% were unidentifiable due to partial visibility, low image quality, or unreliable characteristics and were excluded from the analysis. Representations of empty shells without their living organism inside were also excluded from identification because of their likely provenance from worldwide locations (e.g. ‘Cabinet of curiosities’). Specimen sizes were not measured because the relative lengths and widths of specimens were probably not respected in the paintings, as most still-life artists would draw sketches beforehand and compile them later in their final canvas.

**Figure:**
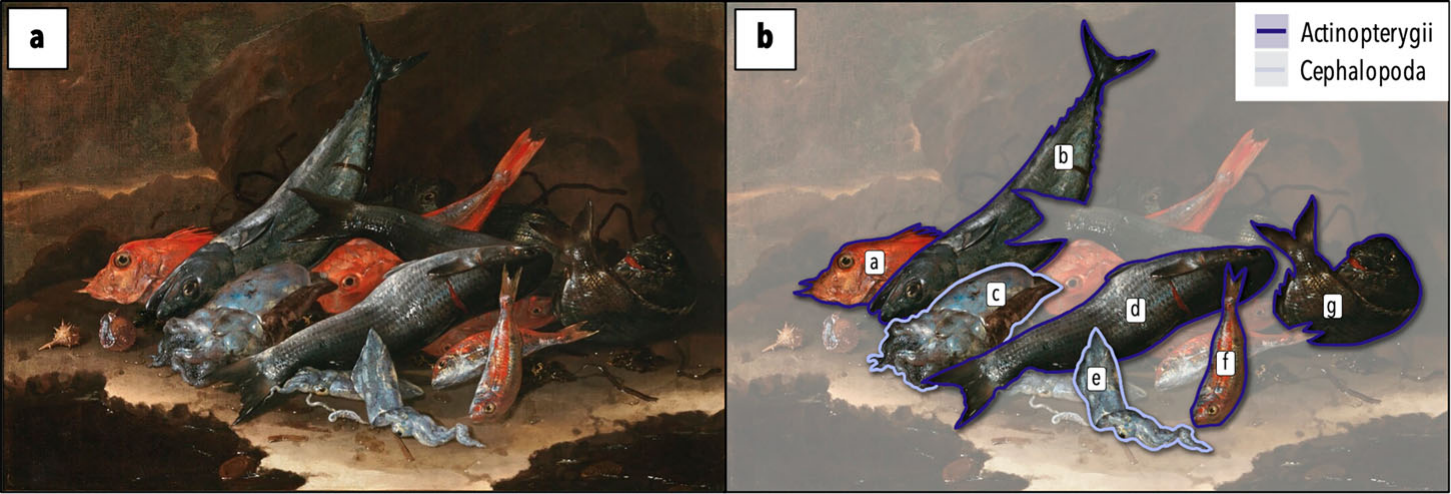
*Box Fig. 1 | Example of taxa identification.* **a** Recco, Giuseppe [Naples, 1634 – Alicante, 1695], *Natura morta con pesci e molluschi* (1675–1680) (oil on canvas, 76 × 102 cm; Collezione Intesa Sanpaolo, Gallerie d’Italia (Naples); image courtesy of the Archivio Patrimonio Artistico Intesa Sanpaolo, photographic credit: Luciano Pedicini, Naples). **b** Same painting with highlights on the taxa identified: **a** piper gurnard (*Trigla lyra*), **b** mackerel (*Scomber scombrus*), **c** common cuttlefish (*Sepia officinalis*), **d** grey mullet (Mugilidae), **e** squid (*Loligo* sp.), **f** red mullet (*Mullus* sp.), **g** gilthead seabream (*Sparus aurata*).

Box 2 Sampling effort Achieving precision in historical data analysis requires significant effort. Randomized accumulation and extrapolation curves can help determine the necessary sample size for biodiversity trends in art. In this study, we analysed 384 Italian still-life paintings from three geographic zones (inland, Adriatic, Liguro-Tyrrhenian) and three time-periods ([1500–1650] [1650–1700] [1700–1800]). The extrapolation of taxonomic richness indicated a detection of about 90%, suggesting that a total sampling size of about 390 paintings would exhaustively detect 95% of taxonomic richness represented in still-life paintings from Italy (Box Fig. 2a). Based on the spatiotemporal selection of paintings, we found that about 134, 86 and 137 paintings should be sampled for the selected time-periods ([1500–1650] [1650–1700] and [1700–1800], respectively) to detect 95% of richness (Box Fig. 2b). Similarly, the number of paintings needed to obtain an exhaustive taxonomic richness also varies across geographic regions, with values ranging from about 93 to 180 paintings (Box Fig. 2c). Considering taxonomic classes, more than 50% of taxonomic richness in all classes can be obtained with a selection of at least 50 paintings, except for Mammalia (30%) and Petromyzonti (24%) (Box Table 1).

These results suggest that the sampling size necessary for identifying trends in biodiversity is acceptably low, which indicates that estimates calculated on statistical analyses can be considered suitable for biodiversity assessment, with rather high confidence.

**Figure:**
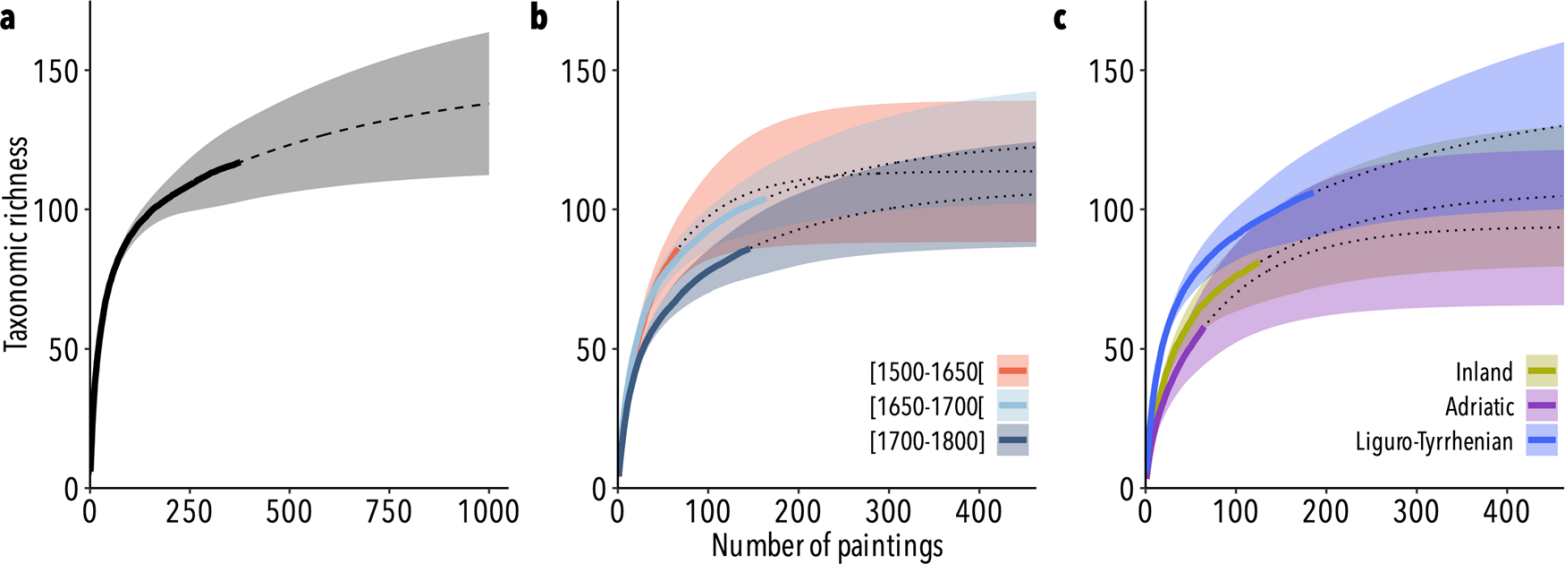
*Box Fig. 2 | Taxonomic richness in paintings.* Randomized accumulation curves based on the interpolation (solid lines) and extrapolation (dotted lines) of taxonomic richness estimated with a 95% confidence interval (shaded regions) based on the number of paintings for **a** all paintings, **b** time-periods and **c** geographic zones.

**Box Table 1 |** Number (*N*) of taxa observed by paintings sampled for each of the taxonomic classes with calculation of the proportion (%) of detected taxa for a selection of 5, 10, 20, 50 and 100 paintings.

**Table:** 

Taxonomic classes	Total *N* of taxa	*N* of taxa observed by number of paintings sampled				
		5	10	20	50	100
Actinopterygii	88	13.05 (15%)	21.20 (24%)	32.75 (37%)	49.38 (56%)	60.30 (69%)
Bivalvia	11	1.73 (16%)	2.87 (26%)	4.36 (40%)	6.14 (56%)	7.48 (68%)
Cephalopoda	3	0.72 (24%)	1.37 (46%)	2.22 (74%)	2.90 (97%)	2.99 (100%)
Echinoidea	2	0.47 (24%)	0.69 (35%)	0.96 (48%)	1.11 (56%)	1.21 (61%)
Elasmobranchii	8	0.65 (8%)	1.10 (14%)	2.03 (25%)	3.86 (48%)	5.46 (68%)
Gastropoda	2	0.23 (12%)	0.44 (22%)	0.71 (36%)	1.17 (59%)	1.44 (72%)
Malacostraca	11	2.76 (25%)	4.02 (37%)	5.47 (50%)	6.98 (63%)	7.96 (72%)
Mammalia	1	0.03 (3%)	0.04 (4%)	0.04 (4%)	0.10 (10%)	0.19 (19%)
Octocorallia	1	0.27 (27%)	0.45 (45%)	0.71 (71%)	0.96 (96%)	0.99 (99%)
Petromyzonti	1	0.03 (3%)	0.04 (4%)	0.13 (13%)	0.24 (24%)	0.57 (57%)
Reptilia	2	0.17 (9%)	0.38 (19%)	0.69 (35%)	1.23 (62%)	1.71 (86%)

## Supplementary information

Supplementary_Information

## Data Availability

No datasets were generated during the current study and the datasets that were analysed are available on demand to corresponding author.

## References

[1] Kittinger, J. N., McClenachan, L., Gedan, K. B. & Blight, L. K. *Marine Historical Ecology in Conservation: Applying the Past to Manage for the Future* (University of California Press, 2015).

[2] Thurstan, R. H. The potential of historical ecology to aid understanding of human–ocean interactions throughout the Anthropocene. *J. Fish Biol.* **101**, 351–364 (2022). 35061243 10.1111/jfb.15000 PMC9545720

[3] Szabó, P. & Hédl, R. Advancing the integration of history and ecology for conservation: history, ecology, and conservation. *Conserv. Biol.* **25**, 680–687 (2011). 21771076 10.1111/j.1523-1739.2011.01710.x

[4] Iglésias, S. P. & Mollen, F. H. L’histoire de la description du squale bouclé *Echinorhinus brucus* (Bonnaterre, 1788) (Echinorhinidae) et la redécouverte des illustrations du type perdu. *Zoosystema* **42**, 173–193 (2020).

[5] Reeves, R. R., Smith, T. D., Josephson, E. A., Clapham, P. J. & Woolmer, G. Historical observations of humpback and blue whales in the North Atlantic Ocean: clues to migratory routes and possibly additional feeding grounds. *Mar. Mammal Sci.* **20**, 774–786 (2004).

[6] Sáenz-Arroyo, A., Roberts, C. M., Torre, J., Cariño-Olvera, M. & Hawkins, J. P. The value of evidence about past abundance: marine fauna of the Gulf of California through the eyes of 16th to 19th century travellers. *Fish Fish.* **7**, 128–146 (2006).

[7] Ferretti, F., Myers, R. A., Serena, F. & Lotze, H. K. Loss of large predatory sharks from the Mediterranean Sea. *Conserv. Biol.* **22**, 952–964 (2008). 18544092 10.1111/j.1523-1739.2008.00938.x

[8] Barrett, J. H. An environmental (pre)history of European fishing: past and future archaeological contributions to sustainable fisheries. *J. Fish Biol.* **94**, 1033–1044 (2019). 30746714 10.1111/jfb.13929

[9] Ames, E. P. Atlantic cod stock structure in the Gulf of Maine. *Fisheries* **29**, 10–28 (2004).

[10] McClenachan, L. Documenting loss of large trophy fish from the Florida Keys with historical photographs. *Conserv. Biol.* **23**, 636–643 (2009). 19183214 10.1111/j.1523-1739.2008.01152.x

[11] Carroll, É. & Faget, D. Histospongia: a database on the Mediterranean sponge (18th century). *Cah. Biol. Mar.* **57**, 317–321 (2016).

[12] Zu Ermgassen, P. S. E. et al. Historical ecology with real numbers: past and present extent and biomass of an imperilled estuarine habitat. *Proc. R. Soc. B Biol. Sci.* **279**, 3393–3400 (2012). 10.1098/rspb.2012.0313 PMC3396889 22696522

[13] Van Houtan, K. S., McClenachan, L. & Kittinger, J. N. Seafood menus reflect long-term ocean changes. *Front. Ecol. Environ.* **11**, 289–290 (2013).

[14] Fourt, M., Faget, D. & Pérez, T. Compilation of multi-source historical data on Mediterranean sponge fisheries. In *Aix-Marseille University, CNRS, IRD and Avignon University**PANGAEA* (CoreTrustSeal, 2017).

[15] Haidvogl, G. et al. Typology of historical sources and the reconstruction of long-term historical changes of riverine fish: a case study of the Austrian Danube and northern Russian rivers. *Ecol. Freshw. Fish* **23**, 498–515 (2014). 25284959 10.1111/eff.12103 PMC4180929

[16] Fortibuoni, T. et al. Fish and fishery historical data since the 19th century in the Adriatic Sea, Mediterranean. *Sci. Data* **4**, 170104 (2017). 28895949 10.1038/sdata.2017.104 PMC5595044

[17] Fossile, T., Ferreira, J., Bandeira, D. D. R., Dias-da-Silva, S. & Colonese, A. C. Integrating zooarchaeology in the conservation of coastal-marine ecosystems in Brazil. *Quat. Int.* **545**, 38–44 (2020).

[18] Pinnegar, J. K. & Engelhard, G. H. The ‘shifting baseline’ phenomenon: a global perspective. *Rev. Fish Biol. Fish.* **18**, 1–16 (2008).

[19] Begossi, A. & Caires, R. Art, fisheries and ethnobiology. *J. Ethnobiol. Ethnomedicine* **11**, 16 (2015). 10.1186/1746-4269-11-17 PMC4506421 26187281

[20] Guidetti, P. & Micheli, F. Ancient art serving marine conservation. *Front. Ecol. Environ.* **9**, 374–375 (2011).

[21] Pollard, D. A. et al. *Epinephelus marginatus*. *The IUCN Red List of Threatened Species.* https://www.iucnredlist.org/ (IUCN, 2018).

[22] Bennema, F. P. & Rijnsdorp, A. D. Fish abundance, fisheries, fish trade and consumption in sixteenth-century Netherlands as described by Adriaen Coenen. *Fish. Res.* **161**, 384–399 (2015).

[23] Overduin-de Vries, A. M. & Smith, P. J. Fishing in the past: Biodiversity, art history, and citizen science – Preliminary results. in *Ichthyology in Context (1500–1880)* (eds. Smith, P. J. & Egmond, F.) 298–321 (E.J. Brill, 2023).

[24] Clavel, B. Un poisson peut en cacher un autre. *Nouv. Archéologie* **148**, 23–27 (2017).

[25] Holm, P. et al. The North Atlantic fish revolution (ca. AD 1500). *Quat. Res.* **108**, 92–106 (2022).

[26] Tribot, A.-S., Faget, D., Villesseche, H., Richard, T. & Changeux, T. Multi-secular and regional trends of aquatic biodiversity in European early modern paintings: toward an ecological and historical significance. *Ecol. Soc.* **26**, art26 (2021).

[27] Depauw, L. et al. The use of photos to investigate ecological change. *J. Ecol.* **110**, 1220–1236 (2022).

[28] Gaynor, A. & McLean, I. The limits of art history: towards an ecological history of landscape art. *Landsc. Rev.* **11**, 4–14 (2005).

[29] Bom, R. A., Van De Water, M., Camphuysen, K. C. J., Van Der Veer, H. W. & Van Leeuwen, A. The historical ecology and demise of the iconic Angelshark *Squatina squatina* in the southern North Sea. *Mar. Biol.* **167**, 91 (2020).

[30] Brander, K. Impacts of climate change on fisheries. *J. Mar. Syst.* **79**, 389–402 (2010).

[31] Burrows, M. T. et al. The pace of shifting climate in marine and terrestrial ecosystems. *Science* **334**, 652–655 (2011). 22053045 10.1126/science.1210288

[32] Lleonart, J. & Recasens, L. Fisheries and the environment in the Mediterranean Sea. *Stud Rev* **66**, 5–18 (1997).

[33] Gombrich, E. H., Combe, J. & Lauriol, C. *Histoire de l’art* (Flammarion, 1994).

[34] Salerno, L. & Wolf, R. E. *La Natura Morta Italiana: 1560–1805* (Ugo Bozzi, 1984).

[35] Russ, H. & Trentacost, A. Wild food in an urban environment: Freshwater fish consumption in the archaic town of Forcello (northern Italy). *Anthropozoologica* **56**, 71–85 (2021).

[36] Volta, P. et al. Fish assemblages in deep Italian subalpine lakes: History and present status with an emphasis on non-native species. *Hydrobiologia* **824**, 255–270 (2018).

[37] De Nicolò, M. L. *Antichie Maniere Di Pescare* (Circolo Nautico Cattolica, 2021).

[38] Tamburini, E. & Turolla, E. The development of oyster farming in Italy: An innovation opportunity for mollusks farming diversification. *J. Aquac. Res. Dev.* **14**, 1–2 (2023).

[39] De Nicolò, M. L. Production et consommation du poisson de mer. L’espace adriatique (XVe-XVIIIe siècle). in *Moissonner la mer. Economies, sociétés et pratiques halieutiques méditerranéennes (XVe-XXIe siècles)* 53–67 (Karthala, 2018).

[40] De Nicolò, M. L. *Del Mangiar Pesce Fresco, ‘Salvato’, ‘Navigato’ Nel Mediterraneo. Alimentazione, Mercato, Pesche Ancestrali (Secc. XIV-XIX)*, Vol. 21 (Rerum maritimarum, 2019).

[41] De Nicolò, M. L. *Pesce Bianco, Pesce Rosa. Cefalo e Fragolino. Storia, Produzione, Tradizioni Alimentari* (Organizzazione produttori di Fano, 2019).

[42] Pitcher, T. J. & Lam, M. E. Fish commoditization and the historical origins of catching fish for profit. *Marit. Stud.* **14**, 2 (2015).

[43] Faget, D. *L’écaille et Le Banc: Ressources de La Mer Méditerranée Moderne. XVIe-XVIIIe Siècle* (Presses universitaires de Provence, 2017).

[44] De Nicolò, M. L. *Dal Banco Di Vendita a Tutte Le Mense. Pesci Molluschi Crostacei Dal Tardo Meioevo Alla Tradizione*, Vol. 22 (Rerum maritimarum, 2020).

[45] Mollat, M. & Adam, P. Histoire des pêches maritimes en France. in *Annales de géographie,* 350–352 (Bibliothèque historique, 1987).

[46] De Nicolò, M. L. Recherches sur l’histoire de la pêche en Méditerranée: Tartanes de Provence, tartanes de Vénétie, trabacs, modèles adriatiques pour la pêche à la traîne et le petit cabotage (XVIIe-XVIIIe siècles). *Cah. Méditerranée* 309–323 (2012).

[47] Ravier, C. & Fromentin, J. M. Long-term fluctuations in the eastern Atlantic and Mediterranean bluefin tuna population. *ICES J. Mar. Sci.* **58**, 1299–1317 (2001).

[48] MacKenzie, B. R. et al. New historical data for long-term swordfish ecological studies in the Mediterranean Sea. *Earth Syst. Sci. Data* **13**, 5867–5877 (2021).

[49] Pagá García, A., Di Natale, A., Tensek, S. & Palma, C. Historical and recent data of Sicilian traps: The complexity of data recovery and interpretation. *Collect. Vol. Sci. Pap. ICCAT* **74**, 2873–2886 (2018).

[50] Hoffmann, R. C. A brief history of aquatic resource use in medieval Europe. *Helgol. Mar. Res.* **59**, 22–30 (2005).

[51] Ageeva, D. *Regulation of Fisheries and Fish Consumption in the Early Modern Venetian Republic* (Central European University, 2022).

[52] Price, L. L. & Narchi, N. E. Ethnobiology of *Corallium rubrum*: protection, healing, medicine, and magic. in *Ethnobiology of Corals and Coral Reefs.**Ethnobiology,* 73–86 (Springer, 2015).

[53] Cattaneo-Vietti, R. et al. An overexploited Italian treasure: past and present distribution and exploitation of the precious red coral *Corallium rubrum* (L., 1758) (Cnidaria: Anthozoa). *Ital. J. Zool.* **83**, 443–455 (2016).

[54] Bronzi, P., Castaldelli, G., Cataudella, S. & Rossi, R. The historical and contemporary status of the European sturgeon, *Acipenser sturio* L., in Italy. in *Biology and conservation of the European Sturgeon* Acipenser sturio *L. 1758: The reunion of the European and Atlantic sturgeons* (eds. Williot, P., Rochard, E., Desse-Berset, N., Kirschbaum, F. & Gessner, J.) 227–241 (Springer Berlin, 2011).

[55] De Nicolò, M. L. *Mangiar Pesce Nell’età Moderna. Diritti Di Pesca, Produzione, Conservazione, Consumo* (Grapho 5, 2004).

[56] Cencini, C. Physical processes and human activities in the evolution of the Po delta, Italy. *J. Coast. Res.* **14**, 774–793 (1998).

[57] Abad, R. *La Conjuration Contre Les Carpes: Enquête Sur Les Origines Du Décret de Dessèchement Des Étangs Du 14 Frimaire an II* (Fayard, 2006).

[58] FAO. *GFCM 2030 Strategy for Sustainable Fisheries and Aquaculture in the Mediterranean and the Black Sea* (FAO, 2021).

[59] Erlandson, J. M. & Rick, T. C. Archaeology meets marine ecology: The antiquity of maritime cultures and human impacts on marine fisheries and ecosystems. *Annu. Rev. Mar. Sci.* **2**, 231–251 (2010). 10.1146/annurev.marine.010908.163749 21141664

[60] Carpentieri, P. *Incidental Catch of Vulnerable Species in Mediterranean and Black Sea Fisheries—A Review* (FAO, 2021).

[61] Scarpa, F., Sanna, D., Azzena, I., Cossu, P. & Casu, M. From dark to light and back again: Is *Pinna nobilis*, the largest mediterranean shellfish, on the brink of extinction?. *Veterinaria* **70**, 1–14 (2021).

[62] Chidichimo, G., Gattuso, C., Biasone, P. & Villella, F. I tesori del mare: La seta e la *Pinna nobilis*. in *Atti del terzo Convegno Internazionale,* Vol. 3, 108–115 (III Conference “Diagnosis, Conservation and Valorization of Cultural Heritage”, 2012).

[63] Lucentini, L. et al. Molecular and phenotypic evidence of a new species of genus Esox (Esocidae, Esociformes, Actinopterygii): The Southern Pike, *Esox flaviae*. *PLoS ONE* **6**, e25218 (2011). 22164201 10.1371/journal.pone.0025218 PMC3229480

[64] Gandolfi, A. et al. Population genetics of pike, genus *Esox* (Actinopterygii, Esocidae), in Northern Italy: Evidence for mosaic distribution of native, exotic and introgressed populations. *Hydrobiologia* **794**, 73–92 (2017).

[65] Casu, M. et al. Appraising the genetic makeup of an allochthonous Southern Pike population: An opportunity to predict the evolution of introgressive hybridization in isolated populations?. *Animals* **13**, 380 (2023). 36766269 10.3390/ani13030380 PMC9913590

[66] Bianco, P. G. An update on the status of native and exotic freshwater fishes of Italy. *J. Appl. Ichthyol.* **30**, 62–77 (2014).

[67] Lorenzoni, M. et al. Cryptic diversity within endemic Italian barbels: Revalidation and description of new *Barbus* species (Teleostei: Cyprinidae). *J. Fish Biol.* **98**, 1433–1449 (2021). 33486760 10.1111/jfb.14688

[68] Bianco, P. G. A revision of the Italian *Barbus* species (Cypriniformes: Cyprinidae). *Ichthyol. Explor. Freshw.* **6**, 305–324 (1995).

[69] Bianco, P. & Ketmaier, V. Anthropogenic changes in the freshwater fish fauna of Italy, with reference to the central region and Barbus graellsii, a newly established alien species of Iberian origin. *J. Fish Biol.* **59**, 190–208 (2001).

[70] Carosi, A., Ghetti, L., La Porta, G. & Lorenzoni, M. Ecological effects of the European barbel *Barbus barbus* (L., 1758) (Cyprinidae) invasion on native barbel populations in the Tiber River basin (Italy). *Eur. Zool. J.* **84**, 420–435 (2017).

[71] Zaccara, S. et al. Genetic and phenotypic displacement of an endemic *Barbus* complex by invasive European barbel *Barbus barbus* in central Italy. *Biol. Invasions* **23**, 521–535 (2021).

[72] Zaccara, S. et al. Morphologic and genetic variability in the *Barbus* fishes (Teleostei, Cyprinidae) of Central Italy. *Zool. Scr.* **48**, 289–301 (2019).

[73] Ferrari, C. et al. Conservation genetics of barbel species (Teleostei, Cyprinidae) facing hybridization and introgression along an elevational gradient in protected areas of northern Italy. *Zool. Scr.* **54**, 1–16 (2024).

[74] Aquiloni, L., Tricarico, E. & Gherardi, F. Crayfish in Italy: Distribution, threats and management. *Int. Aquat. Res.* **2**, 1–14 (2010).

[75] Tsoucalas, G. & Sgantzos, M. Electric current to cure arthritis and cephalaea in ancient Greek medicine. *Mediterr. J. Rheumatol.* **27**, 198–203 (2016).

[76] Karamanlidis, A. Current status, biology, threats and conservation priorities of the vulnerable Mediterranean monk seal. *Endanger. Species Res.* **53**, 341–361 (2024).

[77] Clottes, J., Beltrán, A., Courtin, J. & Cosquer, H. The Cosquer cave on Cape Morgiou. *Marseilles. Antiquity* **66**, 583–598 (1992).

[78] Johnson, W. M. & Lavigne, D. D. *Monk Seals in Antiquity. The Mediterranean Monk Seal (*Monachus Monachus*) in Ancient History and Literature*, Vol. 35 (Netherlands Commission for International Nature Protection, 1999).

[79] Pauly, D. Anecdotes and the shifting baseline syndrome of fisheries. *Trends Ecol. Evol.* **10**, 430 (1995). 21237093 10.1016/s0169-5347(00)89171-5

[80] Wiesner, M. E. *Early Modern Europe, 1450–1789*, Vol. 2 (Cambridge University Press, 2006).

[81] Camuffo, D., Bertolin, C., Schenal, P., Craievich, A. & Granziero, R. The Little Ice Age in Italy from documentary proxies and early instrumental records. *Méditerranée* **122**, 17–30 (2014).

[82] Mensing, S. et al. Human and climatically induced environmental change in the Mediterranean during the medieval climate anomaly and little ice age: a case from central Italy. *Anthropocene* **15**, 49–59 (2016).

[83] Blom, P. *Nature’s Mutiny: How the Little Ice Age of the Long Seventeenth Century Transformed the West and Shaped the Present* (Liveright Publishing, 2019).

[84] De Vries, J. Population. in *Handbook of European History 1400–1600: Late Middle Ages, Renaissance and Reformation* (eds. Brady, T., Oberman & Tracy, J. D.) Vol. 1, XXV–50 (E.J. Brill, 1994).

[85] Kopytoff, I. The cultural biography of things: commoditization as process. in *The social life of things: Commodities in cultural perspective,* 64–91 (Cambridge University Press, 1986).

[86] Jollet, É. *La nature morte ou La place des choses: L’objet et son lieu dans l’art occidental* (Hazan, 2007).

[87] Della Ragione, A. *La Natura Morta Napoletana Del Settecento* (Napoli Arte, 2010).

[88] Bénézit, E. *Dictionnaire des peintres, sculpteurs, dessinateurs et graveurs* (Librairie Gründ, 1939).

[89] Laclotte, M. & Cuzin, J.-P. *Dictionnaire de La Peinture* (Larousse, 1996).

[90] Bianco, P. G. & Delmastro, G. Recenti novità tassonomiche riguardanti i pesci d’acqua dolce autoctoni in Italia e descrizione di una nuova specie di luccio. in *Researches on Wildlife Conservation,* Vol. 2, 1–14 (Elettronico, 2011).

[91] Kottelat, M. & Freyhof, J. *Handbook of European Freshwater Fishes*, Vol. 13 (Publications Kottelat Cornol, 2007).

[92] Rossi, G. et al. Mitochondrial phylogeny and taxonomic revision of Italian and Slovenian fluvio-lacustrine barbels, *Barbus* sp. (Cypriniformes, Cyprinidae). *BMC Zool.* **6**, 8 (2021). 37170173 10.1186/s40850-021-00073-x PMC10127354

[93] Oksanen, J. et al. Vegan: community ecology package 2.7-0 (2024).

[94] R Core Team. R: a language and environment for statistical computing. R foundation for statistical computing (2024).

[95] TAXREF, [Eds]. Référentiel taxonomique pour la France. PatriNat (OFB-CNRS-MNHN-IRD), Muséum national d’Histoire naturelle, Paris. Archive de téléchargement contenant 8 fichiers générés le 10 janvier 2024. *TAXREF v17.0.* https://inpn.mnhn.fr/telechargement/referentielEspece/taxref/17.0/menu (2024).

