# Supplementary material for: Italian still life paintings as a resource for reconstructing past Mediterranean aquatic biodiversity
Source: NPJ Biodivers. 2025 Sep 2;4:33. doi: 10.1038/s44185-025-00103-8 (PMC12405436; doi:10.1038/s44185-025-00103-8)
Supplement: Supplementary file 1 — Supplementary_Information [file 44185_2025_103_MOESM1_ESM.pdf]

Supplementary information: Italian still life paintings as a resource for reconstructing past Mediterranean aquatic biodiversity. Merquiol, L., Tribot, A.-S., Faget, D., Denys, G.P.J., Richard, T., Changeux, T.

Supplementary information checklist

Figure S1 Examples of still-life paintings representing fishing gear. a) Jacob van de Kerckhoven, *Natura morta con pesci, molluschi e crostacei* [circa 1660-1712, active in Venetia from 1683 under the name of Giacomo da Castello]. Credits: Fondazione Federico Zeri, Università di Bologna, photographic credit: anonymous (https://catalogo.fondazionezeri.unibo.it/scheda/opera/88709/Kerckhoven%20Jacob%20van%20de%2C%20Natura%20morta%20con%20pesci%2C%20molluschi%20e%20crostacei), **b)** Francesco Della Questa, *Natura morta di pesci* [circa 1666-1723]. Credits: Fondazione Federico Zeri, Università di Bologna, photographic credit: anonymous (https://catalogo.fondazionezeri.unibo.it/scheda/opera/88583/Della%20Questa%20Francesco%2C%20Natura%20morta%20con%20pesci), **c)** Antonio Tanari, *Pesci* [circa 1610-1630]. Credits: Wikimedia commons, photographic credit: Sailko (https://commons.wikimedia.org/wiki/ File:Antonio_tanari,_pesci,_1610-30_ca..JPG).

Figure S2 Information on artists' cities of activity, number of paintings found per city and spatial analysis of aquatic taxa representation. a) Cities of activity of the studied artists characterised by spatial characteristics (average elevation, distance from the sea, drainage area), demographic variables (historical population), and number of paintings sampled in each city across studied time periods, and **b)** Pearson's residual values of chi-squared tests performed on the proportion (%) of taxa from aquatic environments (freshwater, anadromous, catadromous, marine) and habitats (pelagic, benthopelagic, benthic) across cities (X-squared = 113.39, p-value < 0.001 and X-squared = 25.51, p-value = 0.49, for environments and habitats, respectively). Residual values are indicated by the coloured scale bars, with positive (blue) and negative (red) associations between variables.

Figure S3 Examples of still-life paintings representing cryptic species identified by taxonomy experts. Pike species: **a)** *Esox cisalpinus* in *Natura morta con pesci, crostacei e limone*, Boselli, Felice [circa 1700-1732]. Credits: Fondazione Federico Zeri, Università di Bologna, photographic credit: foto Amoretti (https://catalogo.fondazionezeri.unibo.it/scheda/opera/87140/Boselli%20Felice%2C%20Natura%20morta%20con%20pesci%2C%20crostacei%20e%20limone), **b)** *Esox lucius* in *Natura morta con pesci, crostacei e conchiglie*, Boselli, Felice [circa 1680-1732]. Credits: Fondazione Federico Zeri, Università di Bologna, photographic credit: foto Wells (http://catalogo.fondazionezeri.unibo.it/scheda/opera/87305/Boselli%20Felice%2C%20Natura%20morta%20con%20pesci%2C%20crostacei%20e%20conchiglie). Barbel species: **c)** *Barbus plebejus* in *Natura morta con pesci, crostacei, molluschi, selvaggina, frutta e ortaggi*, Kerckhoven, Jacob van de [circa 1660-1712]. Credits: Fondazione Federico Zeri, Università di Bologna, photographic credit: anonymous (https://catalogo.fondazionezeri.unibo.it/scheda/opera/88742/Kerckhoven%20Jacob%20van%20de%2C%20Natura%20morta%20con%20pesci%2C%20crostacei%2C%20molluschi%2C%20selvaggina%2C%20frutta%20e%20ortaggi), **d)** *Barbus fucini* in *Still life of fish with a char, a bass, a scorpionfish, a sea bream, and shells*, Ruoppolo, Giovan Battista [1670]. Credits: Wikimedia commons, photographic credit: UWCTransferBot (https://commons.wikimedia.org/wiki/File:Giovanni_Battista_Ruoppolo_-_Still_life_of_Fish_with_Char,_Bass,_Rockfish,_sea_Bream_and_Shells.jpg), **e)** *Barbus tyberinus sensus lato* (including *B. samniticus*) in *The fishmonger's shop*, Passerotti, Bartolomeo [1580]. Credits: Wikimedia commons, photographic credit: JarektUploadBot (https://commons.wikimedia.org /wiki/File:Bartolomeo_Passerotti_-_The_Fishmonger%27s_Shop_-_WGA17072.jpg).

Table S1 Results of the indicator taxa analysis were performed on the count of taxa represented in paintings from the identified geographic zones (inland, Adriatic, Liguro-Tyrrhenian). Only significant results are displayed.

Table S2 List of studied painters, active in Italy during the Early Modern Period and whose still-life paintings depict identifiable aquatic organisms. Regions of activity and birth - death dates are indicated when available.

Table S3 List of Mediterranean aquatic taxa identified in the corpus of Italian paintings. Environments associated with each taxon are indicated in brackets (A, anadromous; C, catadromous; F, freshwater; M, marine).

Figure S1 Examples of still-life paintings representing fishing gear. a) Jacob van de Kerckhoven, *Natura morta con pesci, molluschi e crostacei* [circa 1660-1712, active in Venetia from 1683 under the name of Giacomo da Castello]. Credits: Fondazione Federico Zeri, Università di Bologna, photographic credit: anonymous (https://catalogo.fondazionezeri.unibo.it/scheda/opera/88709/Kerckhoven%20Jacob%20van%20de%2C%20Natura%20morta%20con%20pesci%2C%20molluschi%20e%20crostacei), **b)** Francesco Della Questa, *Natura morta di pesci* [circa 1666-1723]. Credits: Fondazione Federico Zeri, Università di Bologna, photographic credit: anonymous (https://catalogo.fondazionezeri.unibo.it/scheda/opera/88583/Della%20Questa%20Francesco%2C%20Natura%20morta%20con%20pesci), **c)** Antonio Tanari, *Pesci* [circa 1610-1630]. Credits: Wikimedia commons, photographic credit: Sailko (https://commons.wikimedia.org/wiki/File:Antonio_tanari,_pesci,_1610-30_ca..JPG).

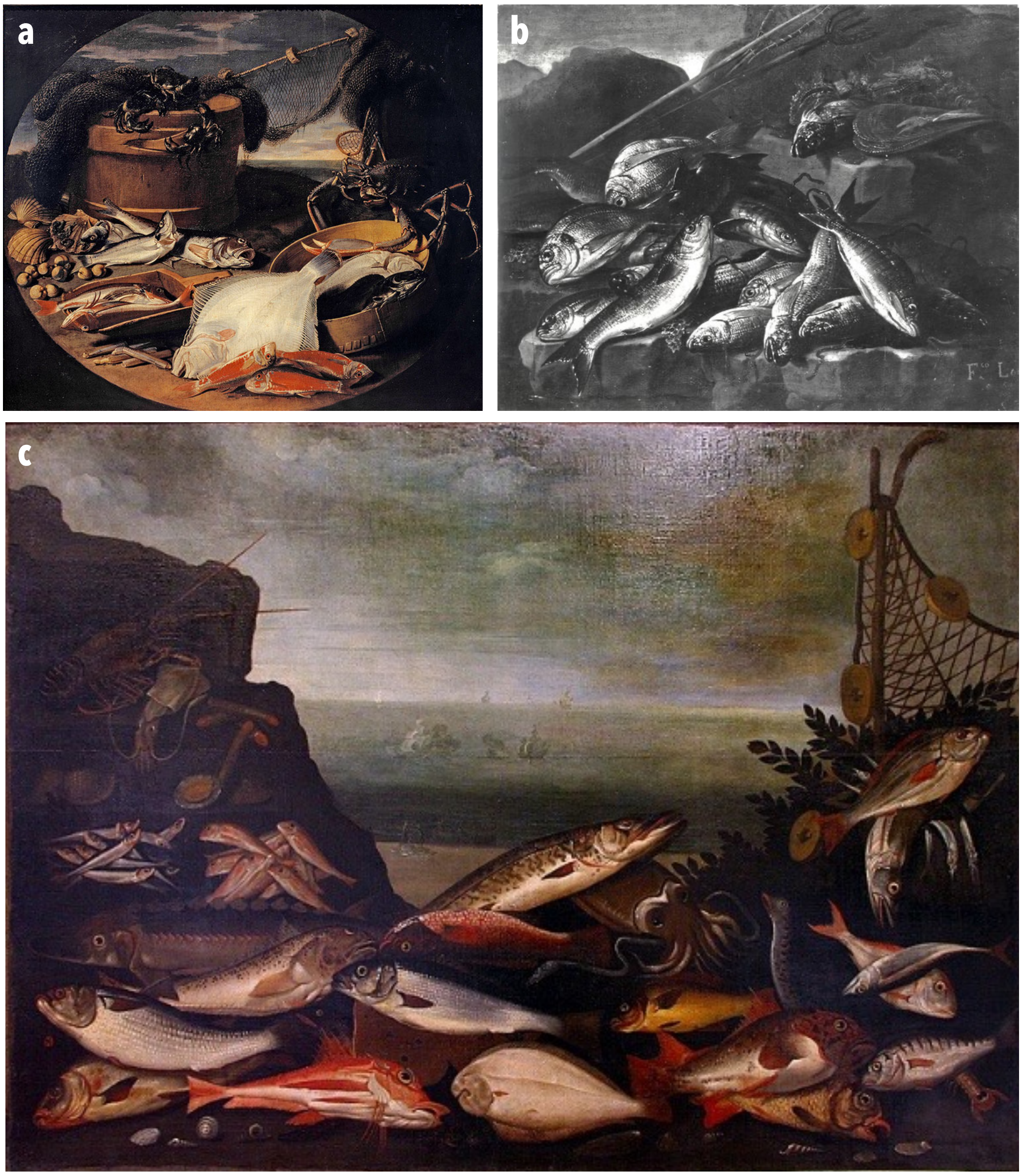

Figure S2 Information on artists' cities of activity, number of paintings found per city and spatial analysis of aquatic taxa representation. a) Cities of activity of the studied artists characterised by spatial characteristics (average elevation, distance from the sea, drainage area), demographic variables (historical population), and number of paintings sampled in each city across studied time periods, and **b)** Pearson's residual values of chi-squared tests performed on the proportion (%) of taxa from aquatic environments (freshwater, anadromous, catadromous, marine) and habitats (pelagic, benthopelagic, benthic) across cities (X-squared = 113.39, p-value < 0.001 and X-squared = 25.51, p-value = 0.49, for environments and habitats, respectively). Residual values are indicated by the coloured scale bars, with positive (blue) and negative (red) associations between variables.

a

City of activity	Average elevation (m)	Distance from the sea (km)	Drainage area of largest river (km²)	Historical population (1500-1800)[1,2]	Number of paintings [1500-1650[	[1650-1700[	[1700-1800]
Bologna	54	75	4,600	55,000 - 71,000	11	-	1
Piacenza	61	85	71,000	17,000 - 33,000	6	18	41
Cremona	85	105	71,000	15,000 - 37,000	5	-	-
Milan	120	120	71,000	69,000 - 135,000	1	1	25
Empoli	28	53	8,200	?	3	-	-
Siena	322	64	3,500	15,000 - 19,000	1	-	-
Fano	12	< 10	1,400	5,000 - 7,000	-	-	5
Faenza	34	38	500	10,000 - 15,000	-	-	3
Rimini	5	< 10	941	8,000 - 13,000	-	-	34
Padua	12	30	2,300	25,000 - 36,000	-	1	3
Venice	9	< 10	2,000	100,000 - 158,000	6	14	4
Genoa	20	< 10	95	60,000 - 91,000	9	6	-
Rome	21	23	17,100	45,000 - 163,000	8	4	1
Naples	65	< 10	715	150,000 - 305,000	17	107	30

b

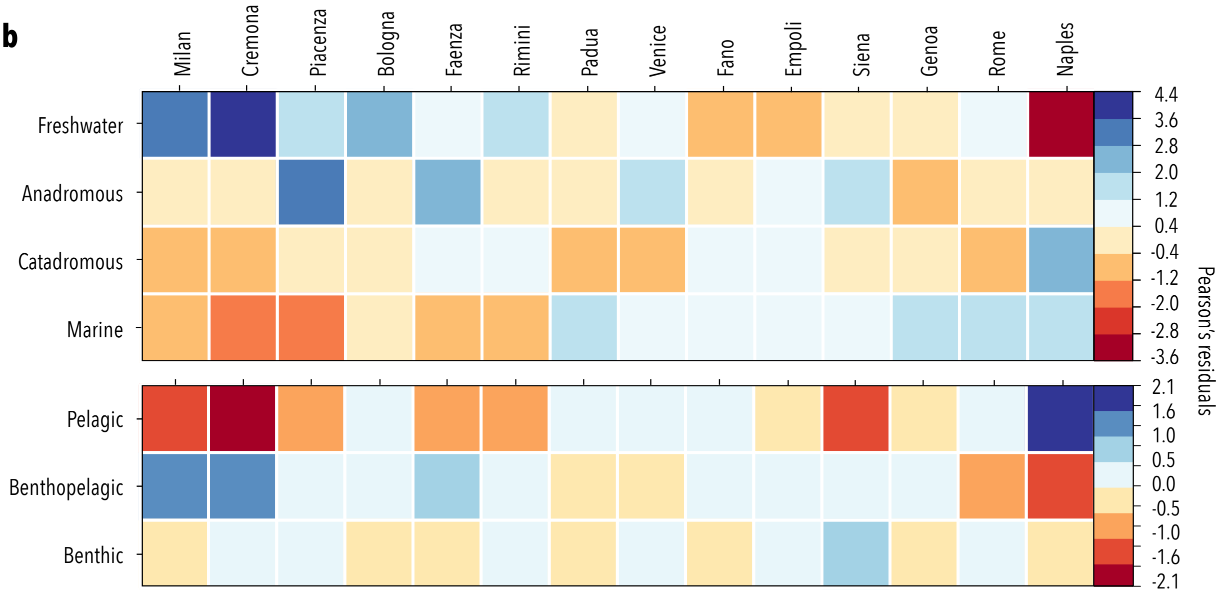

Figure S3 Examples of still-life paintings representing cryptic species identified by taxonomy experts. Pike species: **a)** *Esox cisalpinus* in *Natura morta con pesci, crostacei e limone*, Boselli, Felice [circa 1700-1732]. Credits: Fondazione Federico Zeri, Università di Bologna, photographic credit: foto Amoretti (https://catalogo.fondazionezeri.unibo.it/scheda/opera/87140/Boselli%20Felice%2C%20Natura%20morta%20con%20pesci%2C%20crostacei%20e%20limone), **b)** *Esox lucius* in *Natura morta con pesci, crostacei e conchiglie*, Boselli, Felice [circa 1680-1732]. Credits: Fondazione Federico Zeri, Università di Bologna, photographic credit: foto Wells (http://catalogo.fondazionezeri.unibo.it/scheda/opera/87305/Boselli%20Felice%2C%20Natura%20morta%20con%20pesci%2C%20crostacei%20e%20conchiglie). Barbel species: **c)** *Barbus plebejus* in *Natura morta con pesci, crostacei, molluschi, selvaggina, frutta e ortaggi*, Kerckhoven, Jacob van de [circa 1660-1712]. Credits: Fondazione Federico Zeri, Università di Bologna, photographic credit: anonymous (https://catalogo.fondazionezeri.unibo.it/scheda/opera/88742/Kerckhoven%20Jacob%20van%20de%2C%20Natura%20morta%20con%20pesci%2C%20crostacei%2C%20molluschi%2C%20selvaggina%2C%20frutta%20e%20ortaggi), **d)** *Barbus fucini* in *Still life of fish with a char, a bass, a scorpionfish, a sea bream, and shells*, Ruoppolo, Giovan Battista [1670]. Credits: Wikimedia commons, photographic credit: UWCTransferBot (https://commons.wikimedia.org/wiki/File:Giovanni_Battista_Ruoppolo_-_Still_life_of_Fish_with_Char,_Bass,_Rockfish,_sea_Bream_and_Shells.jpg), **e)** *Barbus tyberinus sensus lato* (including *B. samniticus*) in *The fishmonger's shop*, Passerotti, Bartolomeo [1580]. Credits: Wikimedia commons, photographic credit: JarektUploadBot (https://commons.wikimedia.org/wiki/File:Bartolomeo_Passerotti_-_The_Fishmonger%27s_Shop_-_WGA17072.jpg).

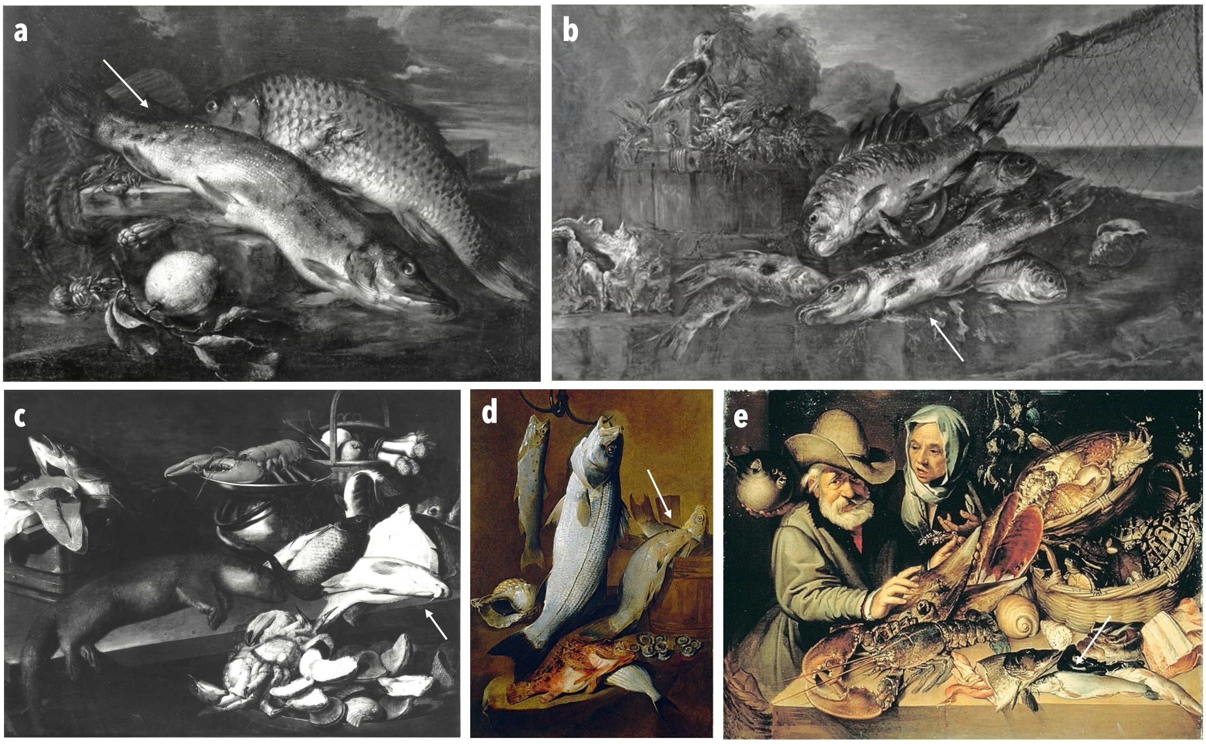

Table S1 Results of the indicator taxa analysis were performed on the count of taxa represented in paintings from the identified geographic zones (inland, Adriatic, Liguro-Tyrrhenian). Only significant results are displayed.

**Geographic zone**	**Statistic**	***p*-value**	
Inland			
Cyprinus carpio	0.39	0.001	***
Esox spp.	0.32	0.001	***
Astacidae	0.20	0.002	**
Carassius sp.	0.16	0.007	**
Barbus spp.	0.16	0.013	*
Salmo trutta	0.12	0.043	*
Adriatic			
Mullus sp.	0.25	0.001	***
Ostrea sp.	0.24	0.001	***
Solea solea	0.24	0.001	***
Pleuronectidae	0.17	0.005	**
Squilla mantis	0.15	0.021	*
Salmo trutta	0.12	0.043	*
Liguro-Tyrrhenian			
Scorpaenidae	0.33	0.001	***
Palinurus elephas	0.28	0.001	***
Sepia officinalis	0.26	0.001	***
Sparus spp.	0.25	0.001	***
Paracentrotus lividus	0.25	0.001	***
Mullus sp.	0.25	0.001	***
Spondylus gaederopus	0.24	0.001	***
Loligo sp.	0.23	0.001	***
Triglidae	0.21	0.001	***
Diplodus spp.	0.21	0.001	***
Mugilidae	0.21	0.002	**
Corallium rubrum	0.21	0.001	***
Scomber spp.	0.20	0.001	***
Lithognathus mormyrus	0.20	0.001	***
Symphodus spp.	0.19	0.003	**
Patella sp.	0.19	0.001	***
Trachurus sp.	0.19	0.003	**
Uranoscopus scaber	0.17	0.004	**
Sardina pilchardus	0.16	0.009	**
Octopus vulgaris	0.15	0.015	*
Belone sp.	0.14	0.020	*
Pagellus sp.	0.14	0.018	*
Pinna nobilis	0.13	0.027	*
Conger conger	0.13	0.043	*

Supplementary information: Italian still life paintings as a resource for reconstructing past Mediterranean aquatic biodiversity. Merquiol, L., Tribot, A.-S., Faget, D., Denys, G.P.J., Richard, T., Changeux, T.

Table S2 List of studied painters, active in Italy during the Early Modern Period and whose still-life paintings depict identifiable aquatic organisms. Regions of activity and birth - death dates are indicated when available.

Artists	Birth – death dates	Artists	Birth – death dates
Campania		*Lazio*	
Anonymous (Naples)	? - ?	Raphael	1483 - 1520
Belvedere, Andrea	1652 - 1732	Salini, Tommaso	1575 - 1625
Bonanni, Andrea	? - ?	Tanari, Antonio	1600 - 1649
Chasse, Barthelemy	1659 - 1720	Valentino, Gian Domenico	1639 - 1715
Cusati, Gaetano	1686 - 1720	*Liguria*	
Della Questa, Francesco	1639 - 1724	Legi, Giacomo	1600 - 1645
Di Caro, Marco	? - ?	Vassallo, Antonio Maria	1617 - 1660
Grosso, Domenico	? - ?	*Lombardy*	
Loth, Onofrio	1665 - 1715	Anonymous (Lombardy)	? - ?
Nani, Mariano	1726 - 1804	Arcimboldo, Giuseppe	1527 - 1593
Porpora, Paolo	1617 - 1673	Blasio, Giovanni	1650 - 1699
Re, Vincenzo	1737 - 1762	Campi, Vincenzo	1536 - 1591
Recco, Elena	1654 - 1715	Cerutti, Giacomo	1698 - 1767
Recco, Giovanni Battista	1615 - 1660	Cipper, Giacomo Francesco	1664 - 1736
Recco, Giuseppe	1634 - 1695	Crastona, Margherita	1696 - 1776
Recco, Nicola Maria	? - ?	Crivelli, Angelo Maria	1703 - 1730
Ruoppolo, Giovan Battista	1629 - 1693	Crivelli, Giovanni	1685 - 1760
Vaccaro, Andrea	1604 - 1670	Pittore di Carlo Torre	1650 - 1700
Emilia-Romagna		*Marche*	
Arboroti, Bartolomeo	1594 - 1676	*Abate, Paolo*	1664 - 1725
Barbieri, Paolo Antonio	1603 - 1649	Ceccarini, Sebastiano	1703 - 1783
Boselli, Felice	1650 - 1732	Magini, Carlo	1720 - 1806
Cittadini, Pier Francesco	1616 - 1681	*Piemonte*	
Crespi, Giuseppe Maria	1665 - 1747	Monfort, Octavianus	1646 - 1696
Levoli, Nicola	1728 - 1801	*Tuscany*	
Monogrammista "PM"	? - ?	Chimenti, Jacopo	1551 - 1640
Passerotti, Bartolomeo	1529 - 1592	Fardella, Giacomo	? - ?
Rivalta, Giovanni	1756 - 1832	Petrazzi, Astolfo	1589 - 1665
Romagnolo	? - ?	*Veneto*	
Soardi, Lodovico	1764 - 1837	Cassana, Giovanni Agostino	1658 - 1720
Lazio		Heintz (II), Joseph	1600 - 1678
Anonymous (Rome)	? - ?	Kerckhoven, Jacob van de	1637 - 1712
Cerquozzi, Michelangelo	1602 - 1660	Strozzi, Bernardo	1581 - 1644
De Pesci, Alessandro	? - ?	Toeput, Lodewijk	1550 - 1605

Supplementary information: Italian still life paintings as a resource for reconstructing past Mediterranean aquatic biodiversity. Merquiol, L., Tribot, A.-S., Faget, D., Denys, G.P.J., Richard, T., Changeux, T.

Table S3 List of Mediterranean aquatic taxa identified in the corpus of Italian paintings. Environments associated with each taxon are indicated in brackets (A, anadromous; C, catadromous; F, freshwater; M, marine).

Family	Taxa
Class: Actinopterygii	
Acipenseridae	*Acipenser* spp. *(A)*
Anguillidae	*Anguilla anguilla (C)*
Belonidae	*Belone* sp. *(M)*
Carangidae	*Trachinotus ovatus (M)*
	Trachurus sp. *(M)*
Clupeidae	*Alosa* sp. *(A)*
	Sardina pilchardus (M)
Congridae	*Conger conger (M)*
Cyprinidae	*Abramis brama (F)*
	Barbus fucini (F)
	Barbus plebejus (F)
	Barbus tyberinus sensus lato (F)
	Barbus sp. *(F)*
	Carassius sp. *(F)*
	Cyprinus carpio (F)
	Leuciscus idus (F)
	Rutilus rutilus (F)
	Squalius cephalus (F)
	Tinca tinca (F)
Engraulidae	*Engraulis* sp. *(M)*
Esocidae	*Esox cisalpinus (F)*
	Esox lucius (F)
	Esox sp. *(F)*
Exocoetidae	*Cheilopogon heterurus (M)*
Haemulidae	*Plectorhinchus mediterraneus (M)*
Labridae	*Coris julis (M)*
	Labrus viridis (M)
	Thalassoma pavo (M)
	Symphodus sp. *(M)*
	Symphodus tinca (M)
	ND *(M)*
Lophiidae	*Lophius* sp. *(M)*
Merluccidae	*Merluccius merluccius (M)*
Molidae	*Mola mola (M)*
Moronidae	*Dicentrarchus labrax (C)*
Mugilidae	ND *(C)*
Mullidae	*Mullus* sp. *(M)*
Muraenidae	*Muraena helena (M)*
Percidae	*Perca fluviatilis (F)*
Pleuronectidae	ND *(M)*
Sparidae	*Pagellus* sp. *(M)*
	Pagrus sp. *(M)*
	Sarpa salpa (M)
	ND *(M)*
Sphyraenidae	*Sphyraena* sp. *(M)*
Syngnathidae	*Hippocampus guttulatus (M)*
Trachinidae	*Trachinus radiatus (M)*
	ND *(M)*
Triglidae	*Chelidonichthys cuculus (M)*
	Chelidonichthys lucerna (M)
	Trigla lyra (M)
	ND *(M)*
Uranoscopidae	*Uranoscopus scaber (M)*
Xiphiidae	*Xiphias gladius (M)*
Zeidae	*Zeus faber (M)*
Class: Bivalvia	
Cardiidae	ND *(M)*
Mytilidae	*Lithophaga lithophaga (M)*
	Mytilus sp. *(M)*
Ostreidae	*Ostrea* sp. *(M)*
Pectinidae	*Pecten maximus (M)*
Pharidae	*Ensis siliqua (M)*
Pinnidae	*Pinna nobilis (M)*
Spondylidae	*Spondylus gaederopus (M)*
Veneridae	ND *(M)*
Class: Cephalopoda	
Loliginidae	*Loligo* sp. *(M)*
Octopodidae	*Octopus vulgaris (M)*
Sepiidae	*Sepia officinalis (M)*
Class: Echinoidea	
Asteriidae	*Marthasterias glacialis (M)*
Parechinidae	*Paracentrotus lividus (M)*
Class: Elasmobranchii	
Rajidae	*Raja clavata (M)*
	ND *(M)*
Torpedinidae	*Torpedo torpedo (M)*
Triakidae	*Mustelus* sp. *(M)*
Scyliorhinidae	*Scyliorhinus* sp. *(M)*
Squalidae	*Squalus acanthias (M)*
	Squalus sp. *(M)*
Squatinidae	*Squatina squatina (M)*

Table S3 Continued.

Family	Taxa	Family	Taxa
Salmonidae	Coregoninae *(F)*	Class: Gastropoda	
	Salmo trutta (A)	Haliotidae	*Haliotis* sp. *(M)*
Sciaenidae	*Argyrosomus regius (M)*	Patellidae	*Patella* sp. *(M)*
	Umbrina cirrosa (M)	Class: Malacostraca	
Scombridae	*Sarda sarda (M)*	Astacidae	ND *(F)*
	Scomber colias (M)	Brachyura	ND *(M)*
	Scomber scombrus (M)	Calappidae	*Calappa* sp. *(M)*
	ND *(M)*	Caridea	ND *(M)*
	Thunnus thynnus (M)	Nephropidae	*Homarus gammarus (M)*
Scorpaenidae	*Scorpaena porcus (M)*	Majidae	*Maja* sp. *(M)*
	Scorpaena scrofa (M)	Palinuridae	*Palinurus elephas (M)*
	ND *(M)*	Potamidae	*Potamon fluviatile (F)*
Sebastidae	*Sebastes* sp. *(M)*	Scyllaridae	*Scyllarides latus (M)*
Serranidae	*Epinephelus* sp. *(M)*	Squillidae	*Squilla mantis (M)*
	Serranus scriba (M)	Class: Mammalia	
Soleidae	*Solea solea (M)*	Mustelidae	*Lutra lutra (F)*
Sparidae	*Sparus aurata (M)*	Class: Octocorallia	
	Spondyliosoma cantharus (M)	Coralliidae	*Corallium rubrum (M)*
	Boops boops (M)	Class: Petromyzonti	
	Diplodus sargus (M)	Petromyzontidae	*Petromyzon marinus (A)*
	Diplodus vulgaris (M)	Class: Reptilia	
	Diplodus sp. *(M)*	Cheloniidae	*Caretta caretta (M)*
	Lithognathus mormyrus (M)		ND *(M)*

References

1. De Vries, J. Population. in *Handbook of European History 1400-1600: Late Middle Ages, Renaissance and Reformation* (eds. Brady, T., Oberman & Tracy, J. D.) vol. 1 XXV–50 (E.J. Brill, Leiden, USA, 1994).
2. Bairoch, P., Batou, J. & Chèvre, P. *La Population Des Villes Européennes de 800 à 1850: Banque de Données et Analyse Sommaire Des Résultats*. vol. 2 (Droz, Genève, Switzerland, 1988).
